# Relaxation Polyamorphism of Rosuvastatin and Physicochemical Characterisation of a Rosuvastatin–Carvedilol Co-Amorphous System

**DOI:** 10.3390/pharmaceutics18091076

**Published:** 2026-08-27

**Authors:** Agata Olszewska, Maria Brycka, Anita Umerska, Lidia Tajber, Marek Pyda, Marcin Skotnicki

**Affiliations:** 1 Industrial Pharmacy Division, Chair and Department of Pharmaceutical Technology, Poznan University of Medical Sciences, Rokietnicka 3, 60-806 Poznan, Poland; agata.olszewska@ump.edu.pl (A.O.);; 2 Doctoral School, Poznan University of Medical Sciences, Bukowska 70, 60-812 Poznan, Poland; 3 School of Pharmacy and Pharmaceutical Sciences, Trinity College Dublin, The University of Dublin, College Green, Dublin 2, D02 PN40 Dublin, Ireland; umerskam@tcd.ie (A.U.); lidia.tajber@tcd.ie (L.T.); 4 Department of Chemistry, University of Tennessee, Knoxville, TN 37996-1600, USA; mpyda@utk.edu; 5 Chemical Sciences Division, Oak Ridge National Laboratory, Oak Ridge, TN 37831-6197, USA

**Keywords:** rosuvastatin, solid-state pharmaceuticals, co-amorphous systems, amorphous solids, relaxation polyamorphism, sorption isotherms

## Abstract

**Objective**: In this study, the solid-state properties of two amorphous forms of rosuvastatin calcium (ROS) and co-amorphous systems of rosuvastatin with carvedilol (CAR) were investigated. **Methods**: The studied systems were investigated using TGA, DSC, TMDSC, PXRD, SAXS, SSNMR, and DVS. **Results**: DSC, TMDSC and SAXS revealed subtle differences between as-received ROS (ROS AR) and freshly prepared amorphous ROS (ROS AM), providing evidence of relaxation polyamorphism. Analysis of the scanning-rate dependence of the glass transition temperature classified ROS as a fragile glass former. Water sorption studies of ROS AR using Brunauer–Emmett–Teller analysis, the Guggenheim–Anderson–de Boer equation, and Young–Nelson models indicated a moderate specific surface area and multilayer adsorption, with water binding both at the surface and within the bulk. Co-amorphous CAR–ROS systems in various molar ratios exhibited single glass transitions, confirming the formation of homogenous amorphous phases. Negative deviations from the Couchman–Karasz equation indicated non-ideal mixing behaviour. Physical ageing studies showed high stability of the ROS AM and CAR-ROS 1:1 co-amorphous system, which remained amorphous after three years of storage. **Conclusions**: Overall, ROS forms stable amorphous and co-amorphous systems with CAR, exhibiting favourable miscibility, structural stability, and potential for application in fixed-dose combination formulations.

## 1. Introduction

Since their introduction to the market, statins have been one of the most commonly prescribed groups of drugs across the world [1]. They inhibit hydroxymethyl glutaryl coenzyme A reductase (HMG-CoA), which leads to a reduction in total cholesterol and LDL blood levels [2]. Elevated LDL cholesterol is a major risk factor for the development of atherosclerosis and, consequently, cardiovascular diseases (CVDs). Despite the widespread use of statins, CVDs remain the main cause of death and disability in Europe [3], highlighting the need for a better understanding and further development of therapies used in CVDs treatment.

Rosuvastatin is commercially available as the calcium salt, which exists in several solid-state forms, including four crystalline polymorphs (**A**, **B**, **B-1** and **C**) and one amorphous form. Form **A** is an anhydrous crystalline compound, whereas form **B** and **C** are hydrated crystalline compounds. Form **B-1** is obtained by dehydrating form **B** [4,5]. Compared with form **A**, the hydrated forms (**B** and **C**) are described as being more soluble in water due to particle size (the median size is between 1 and 50 µm, preferably between 1 and 10 µm), while exhibiting better physical and chemical stability during storage and processing than the amorphous form of rosuvastatin calcium [4].

Amorphous solids are often hygroscopic, meaning they readily absorb moisture from the environment [6]. Water interaction with these solids is characterised by significant uptake into the solid structure; more than is predicted for adsorption to the dry external surface area and specific surface area [6]. However, the presence of water molecules can promote recrystallisation of these materials. This is an important consideration during both the production and storage processes [7]. To study how water molecules interact with the surface and bulk of a substance in its amorphous state, dynamic vapor sorption (DVS) can be used [8].

However, despite their advantageous biopharmaceutical properties, amorphous solids are thermodynamically unstable and tend to recrystallise during storage. To overcome stability limitations, several formulation strategies have been developed to reduce molecular mobility and suppress recrystallisation of amorphous solids. In the last two decades, there has been considerable interest expressed by both academia and industry regarding improving the dissolution rate and stability of APIs by combining amorphous drugs into a single-phase system called a co-amorphous system [9,10], defined as *a multi-component single-phase amorphous solid system which lacks periodicity in lattice and is associated with weak and discrete intermolecular interactions between the components* [11]. It has been found that the use of co-amorphous systems improves physical stability, dissolution rate, and bioavailability compared to amorphous form of single APIs [9,10,11,12,13,14,15,16,17]. The following phenomena have been suggested as potentially responsible for the enhanced stability: an anti-plasticisation effect (elevation of glass transition temperature) and reduced molecular mobility, as well as intermolecular interactions between the APIs in the system [11,12,15,18,19,20,21,22]. Co-amorphisation of rosuvastatin has already been reported with pioglitazone [23]. Co-crystallisation of rosuvastatin has been the most commonly used method to modify its properties and has been carried out with *L*-asparagine and *L*-glutamine [24], and ascorbic acid [25].

Amorphous materials may exhibit different physicochemical properties depending on their previous thermal history and time of storage due to physical ageing [26,27,28,29,30], and thus amorphous glasses can exist in different kinetic states—the term *pseudopolyamorphism* or *relaxation* (*kinetic*) *polyamorphism* has been used to describe this phenomenon [28]. The physical ageing or annealing in the amorphous material, i.e., structural relaxation towards thermodynamic equilibrium as a function of time, is of great importance from the point of view of pharmaceutical sciences and industry [31]. A sample stored below glass transition temperature (*T*_g_) remains in a non-equilibrium state and undergoes physical ageing. At low temperatures, apparent equilibrium is difficult to achieve as the molecular mobility is low, and thus the time for observing physical ageing may be prolonged. Moreover, samples with distinct apparent equilibria existing below *T*_g_ may exhibit different physicochemical properties [32]. Although this phenomenon remains poorly understood, polyamorphism or pseudopolyamorphism [33,34] has been reported for several compounds, including valsartan [29,30], hydrochlorothiazide [35], olaparib [36] and *D*-mannitol [37].

In addition to *T*_g_, fragility is another parameter used to describe the molecular mobility and stability of amorphous materials. The amorphous systems can be classified as either “strong” or “fragile” based on the temperature dependence of mean molecular relaxation time [38,39]. The strong glass shows Arrhenius-type behaviour with their activation energy (*E*_a_) being independent of temperature. Strong glass-formers thus exhibit resistance to structural changes induced with increasing temperature. In contrast, fragile glass shows non-Arrhenius-type behaviour, and its *E*_a_ is dependent on temperature. Fragile liquids are characterised by an unstable structure at their *T*_g_ which tends to reorganise with little thermal provocation into structures fluctuating over a variety of orientations [38].

The qualitative and quantitative characterisation of miscibility, phase separation, crystalline/amorphous nature, molecular mobility, molecular interaction and morphology require advanced analytical methods. The amorphous and co-amorphous systems have been mainly characterised by thermal (Differential Scanning Calorimetry), spectroscopic (Broadband Dielectric Spectroscopy, Fourier-transform Infrared Spectroscopy, Raman Spectroscopy, Terahertz Spectroscopy, Solid-state Nuclear Magnetic Resonance) and diffractometric methods (Powder X-ray diffractometry) [9,40]. Despite a wide variety of analytical methods, the structural characterisation of subtle differences between different amorphous forms, especially obtained by different methods or when physically aged, remains challenging.

This study aimed to characterise selected physicochemical properties of amorphous rosuvastatin and a co-amorphous system of rosuvastatin with carvedilol as a potential fixed-dose combination drug (FDC). The simultaneous administration of both APIs has the potential to lower the nephrotoxicity of solidum valproate [41]. Moreover, it exhibits cardioprotective properties during treatment with doxorubicin [42] and in the case of myocardial infarction [43]. Both drugs are used in the treatment of CVDs, and both are poorly soluble drugs classified as Biopharmaceutical Classification System (BCS) class II compounds. Amorphous rosuvastatin has a relatively high *T*_g_ above 120 °C, which may be used to elevate the low *T*_g_ (ca. 38 °C) of carvedilol in a co-amorphous combination.

## 2. Materials and Methods

### 2.1. Materials

**Figure 1:**
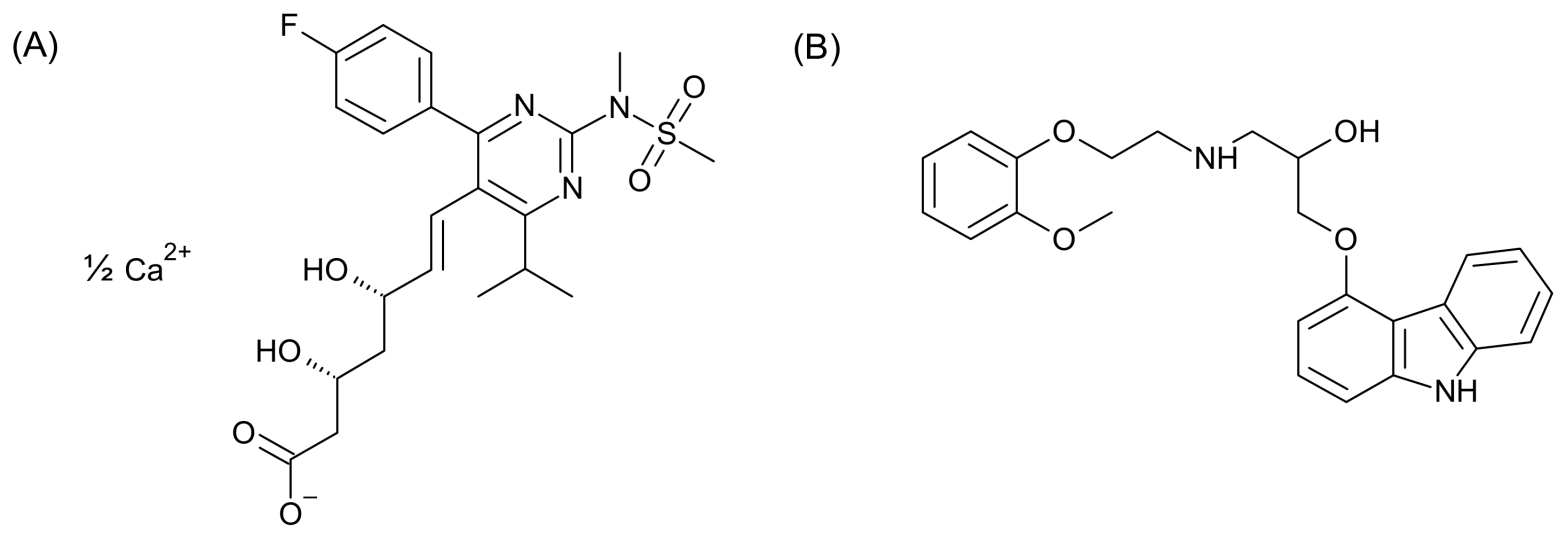
Chemical structure of (**A**) rosuvastatin calcium (ROS), monocalcium bis (+) 7-[4-(4-fluorophenyl)-6-isopropyl-2-(*N*-methyl-*N*-methyl-sulfonylaminopyrimidin)-5-yl]-(3*R*,5*S*)-dihydroxy-(*E*)-6-heptenoate) and (**B**) carvedilol (CAR), 1-(9*H*-carbazol-4-yloxy)−3-{[2s-(2-methoxyphenoxy)ethyl]amino}−2-propanol.

Rosuvastatin calcium (ROS AR, as received; pharmaceutical grade; Figure 1A) was obtained from Biofarm Sp. z o.o. (Poznan, Poland), and used without further treatment. The as-received form was found to be amorphous based on the PXRD results. Fresh amorphous ROS (form AM) was prepared immediately before experimental measurements in the DSC pan in the instrument, or on the plate by heating the sample on the aluminium foil to 150–160 °C, holding for 15–20 min, then cooling to room temperature at a 5 °C·min^−1^ cooling rate. The thermal stability of rosuvastatin was investigated by TGA. The examined sample was stable in a liquid state above the glass transition, showing less than 1% loss of mass at 150 °C held for 50 min and at 160 °C for 15 min.

Carvedilol (CAR CR; crystalline; pharmaceutical grade; Figure 1B) was obtained from Pharmaceutical Research Institute (Warsaw, Poland) and used without further treatment. Its amorphous form (form AM) was prepared immediately before experimental measurements in the DSC pan in the instrument, or on the plate by heating the sample on the aluminium foil to 150–160 °C, holding for 10 min, then cooling to room temperature at about a 5 °C·min^−1^ cooling rate. The material is chemically stable in the liquid state above the melting transition showing less than 0.3% loss of mass at 180 °C held for 60 min.

Physical mixtures of carvedilol/rosuvastatin in different concentrations, i.e., 1:1, 1:2, and 2:1 (mol/mol) and 1:1 (*w*/*w*) were prepared by mixing APIs in a glass mortar for 20 min. Co-amorphous samples were produced by the heating to 150 °C of binary mixtures in situ in the DSC furnace and cooling to −20 °C with at least a 10 °C·min^−1^ cooling rate, or on the plate by heating the sample on the aluminium foil to 150–160 °C, holding for 15–20 min, then cooling to room temperature at a 5 °C·min^−1^ cooling rate.

### 2.2. Thermogravimetric Analysis (TGA)

TGA curves were obtained using a Discovery TGA 5500 (TA Instruments—Waters LLC; New Castle, DE, USA) under a nitrogen gas flow 25 mL·min^−1^. Mass of 1–10 mg of powder samples was placed in an opened platinum pan and heated at a rate of 10 °C·min^−1^ from 30 to 700 °C.

### 2.3. Differential Scanning Calorimetry (DSC) and Temperature-Modulated Differential Scanning Calorimetry (TMDSC)

DSC curves were obtained using a Discovery Q2500 (TA Instruments—Waters LLC; New Castle, DE, USA) (V9.9 Build 303) under a nitrogen gas flow of 50 mL·min^−1^. Sample powders (1–10 mg) were crimped in a T_zero_ hermetic aluminium pan with a hole and heated from −20 to 150 °C. Next, the samples were cooled down with a 10 °C·min^−1^ rate to room temperature and reheated to 140 °C in a second run.

TMDSC curves were obtained using the Discovery Q2500 (TA Instruments—Waters LLC; New Castle, DE, USA) (V9.9 Build 303) with an underlying heating rate 1 °C·min^−1^ and a temperature modulation with amplitude of 0.5 °C and period of 60 s from −50 to 140 °C. The samples were then cooled at a 10, 20 or 50 °C min^−1^ cooling rate to −50 °C without modulation and reheated to 140 °C in a second run.

The enthalpy and temperature were calibrated with indium (m. p. = 156.65 °C, Δ_fus_*h* = 28.45 J·g^−1^), and at least two tests (mostly five) were run on each sample. Melting, crystallisation and relaxation events are quoted as an onset temperature. The heat of fusion for crystalline CAR was integrated with sigmoidal baseline type. Glass transition temperatures are quoted as midpoints except for the calculation of the activation energy of structural relaxation and the fragility parameter. All values were determined using Trios v4.4.0 (TA Instruments—Waters LLC; New Castle, DE, USA) software. Errors are quoted as one standard deviation.

To perform the physical ageing study by DSC, samples of the CAR AM, ROS AM and CAR-ROS co-amorphous systems were heated to 120 °C and held for 5 min to eliminate the effect of prior thermal history and then cooled at 25 °C·min^−1^ below the glass transition temperature to the ageing temperature, *T*_a_, which was 30 °C or 45 °C. The samples were maintained at *T*_a_ for specified times ranging from 0 to 24 h, and then cooled at 25 °C·min^−1^ to 25 °C. Additionally, the samples were annealed in an incubator for over three years at 45 °C.

The activation energy of structural relaxation and the fragility parameter of ROS was determined based on the heating rate dependence of *T*_g_. The amorphous samples were heated to 120 °C to delete the thermal history and then cooled to −20 °C at 10 °C·min^−1^, followed by reheating at the following rates: 1, 2.5, 5, 10, 15, 20 °C·min^−1^. The extrapolated onset of *T*_g_ was used for the calculation of the activation energy of structural relaxation (*E*_a_) and the fragility parameter (*m*) [44,45] represented in Equations (1) and (2):

(1) $$-\frac{E_{a}{(T}_{g})}{R}=\frac{d(\ln q)}{d({T_{g}}^{-1})}$$

(2) $$m=\frac{E_{a}{(T}_{g})}{2.303\times R\times T_{g}}$$

where R is the gas constant, $T_{g}$ is the onset of the glass transition temperature in kelvin and q is the heating rate. DSC curves were obtained using a Discovery Q2500 (TA Instruments—Waters LLC; New Castle, DE, USA) under a nitrogen gas flow of 50 mL·min^−1^.

The thermal behaviour of multi-component amorphous materials in the glass transition region *T*_g_ is described by the phenomenological approach using the Couchman–Karasz equation (CK, Equation (3)) [46]:

(3) $$T_{g12}=\frac{w_{1}T_{g1}+{Kw}_{2}T_{g2}}{w_{1}+{Kw}_{2}}$$

where $T_{g12}$ is the glass transition temperature of a multi-component amorphous material, $T_{g1}$ and $T_{g2}$ are the glass transition temperatures of pure components, and $w_{1}$ and $w_{2}$ are their respective weight fractions. K constant can be expressed as Equation (4):

(4) $$K=\frac{{\Delta c}_{p2}}{{\Delta c}_{p1}}$$

where ${\Delta c}_{p1}$ and ${\Delta c}_{p2}$ represent the changes in heat capacity at *T*_g_ of the pure components.

### 2.4. Powder X-Ray Diffractometry (PXRD)

The PXRD patterns were obtained with a Bruker D8 ADVANCE (Bruker AXS GmbH, Karlsruhe, Germany) or a Rigaku Miniflex II desktop X-ray diffractometer (Rigaku Corporation, Tokyo, Japan) coupled with Cu-Kα radiation (*λ* = 1.5406 Å). Samples were loaded in an aluminium sample holder and levelled with a glass slide. Measurements were conducted in the 2*θ* range of 4–45° at a step scan interval of 0.02 degrees per step and a counting time of 2 s per step. The measurements were performed at room temperature. The obtained data were analysed using the Kalvados program (Karel Knizek, Prague, Czech Republic).

### 2.5. Small Angle X-Ray Scattering (SAXS)

Small angle X-ray scattering profiles were collected on the Bruker Nanostar U device (Bruker AXS GmbH, Karlsruhe, Germany) equipped with an Incoatec IμS anode X-ray source at the wavelength of 1.25 Å and Vantec 2000 detector. A sample measurement cell was positioned at a distance of 35 cm from the detector, allowing the collection of data up to 10 degrees of the scattering angle. The detection was performed in air atmosphere.

### 2.6. Solid-State Nuclear Magnetic Resonance (SSNMR)

Solid-state ^13^C nuclear magnetic resonance spectra were recorded on Bruker Avance HD 400 NMR (Billerica, MA, USA) and Agilent 800 DD2 (Santa Clara, CA, USA) spectrometers operating at ^13^C frequencies of 100.61 and 201.12 MHz. A magic-angle spinning (MAS) probe using 3.2 mm diameter zirconia rotors was used. Typical operating conditions used cross-polarisation (CP) for excitation of the ^13^C magnetisation, with C contact time of 1 or 3 ms (respectively, amorphous and crystalline samples), a recycle delay of 4 s, 512 transients and a spin rate of 20 kHz at temperature 20 °C. Carbon chemical shifts were referenced to the signal for tetramethylsilane via a replacement sample of solid adamantane (38.4 ppm for the high-frequency line).

### 2.7. Dynamic Vapour Sorption (DVS)

DVS studies were carried out using an Advantage-1 automated gravimetric vapor sorption analyser (Surface Measurement Systems Ltd., London, UK). The temperature was maintained at 25.0 ± 0.1 °C. The samples were equilibrated at 0% relative humidity (RH) until a constant mass was obtained (d*m*/d*t* ≤ 0.002 mg/min). The reference mass was recorded, and sorption−desorption analysis was then carried out between 0 and 90% RH, in steps of 10% RH. At each stage, the sample mass was equilibrated (d*m*/d*t* ≤ 0.002 mg/min for at least 10 min) before the RH was changed. An isotherm was calculated from the complete sorption and desorption profile.

To determine the specific surface area of the rosuvastatin sample, Brunauer–Emmett–Teller (BET) analysis was employed Equation (5) [47]. The usual form of the BET equation is as follows:

(5) $$\frac{v}{v_{m}}=\frac{c(\frac{p}{p_{0}})}{\left[1-\left(\frac{p}{p_{0}}\right)\right]\left[1+\left(c-1\right)\left(\frac{p}{p_{0}}\right)\right]}$$

where v is the total adsorbed gas, $v_{m}$ is the gas adsorbed in a monolayer, p is the partial pressure of adsorbate gas, $p_{0}$ is the saturation pressure of adsorbate gas, and *c* is the BET constant Equation (6).

(6) $$c=e^{-(E_{1}-E_{L})/RT}$$

where $E_{1}$ is the heat of sorption for the first layer, $E_{L}$ is the heat of sorption for the multilayer, R is the gas constant, and T is the absolute temperature. However, the BET equation is often used in its linear form Equation (7):

(7) $$\frac{1}{v\left[\left(\frac{p}{p_{0}}\right)-1\right]}=\frac{\left(c-1\right)}{v_{m}c}\left(\frac{p}{p_{0}}\right)+\frac{1}{v_{m}c}$$

The Guggenheim–Anderson–de Boer (GAB) Equation (8) is an extension of the BET equation and is primarily used to describe the adsorption and desorption of water vapours [48,49,50]:

(8) $$W=\frac{W_{m}C_{G}K(\frac{p_{w}}{p_{w0}})}{\left[1-K\left(\frac{p_{w}}{p_{w0}}\right)\right]\left[1-K\left(\frac{p_{w}}{p_{w0}}\right)+W_{m}C_{G}K\left(\frac{p_{w}}{p_{w0}}\right)\right]}$$

where W is the weight of water sorbed per gram of dry sample, $W_{m}$ is the weight of water in the monolayer per gram of dry sample, and $\frac{p_{w}}{p_{w0}}$ is the relative vapour pressure of water. $C_{G}$ Equation (10) and K Equation (9) are the parameters related to the heat of sorption. Parameter $C_{G}$ is a constant related to the difference between free enthalpy of water molecules in a liquid state and in the monolayer and parameter K accounts layer or multilayer of adsorbed water with a degree of binding intermediate to those of the monolayer and the bulk water:

(9) $$K=B\times e^{(H_{L}-H_{m})/RT}$$

(10) $$C_{G}=D\times e^{(H_{1}-H_{m})/RT}$$

where B and D are constant. $H_{L}$ equals to the heat of condensation, $H_{m}$ to the heat of sorption of water in the multilayer, and $H_{1}$ to the heat of sorption of water in the first monolayer.

The Young and Nelson equations were used to describe the equilibrium of water vapour’s sorption and desorption as a function of RH. This model assumes three mechanism of water updates: the formation of a monomolecular layer on the outer surface of the substance Equation (11), formation of a multimolecular layer on top of the first layer Equation (12) and absorption of water into the bulk of the material Equation (13) [51]:

(11) $$\theta =\frac{p_{w}}{p_{w0}}/[\frac{p_{w}}{p_{w0}}+\left(1+\frac{p_{w}}{p_{w0}}\right)E]$$

(12) $$\psi =\frac{p_{w}}{p_{w0}}\times \theta$$

(13) $$\beta =\frac{E\times \frac{p_{w}}{p_{w0}}}{E-\left(E-1\right)\frac{p_{w}}{p_{w0}}}+\frac{E^{2}}{E-1}\ln\left[\frac{E-\left(E-1\right)\frac{p_{w}}{p_{w0}}}{E}\right]-\left(E+1\right)\ln\left(1-\frac{p_{w}}{p_{w0}}\right)$$

where θ is the fraction of the surface that is covered by a layer of adsorbed water molecules, ψ is the fraction of the surface covered by many layers of adsorbed water molecules, and β is the total amount of adsorbed water in multilayers.

Two equations were derived to describe total water sorption, which describes the total absorbed moisture during the sorption process $M_{s}$ Equation (14) and the total absorbed moisture during the desorption process $M_{d}$ Equation (15).

(14) $$M_{s}=A\left(\theta +\beta \right)+B\times \psi$$

(15) $$M_{d}=A\left(\theta +\beta \right)+B\times \theta \times {RH}_{max}$$

where ${RH}_{max}$ is equal to maximum RH, and A Equation (16), B Equation (17) and E Equation (18) are parameters related to the material.

(16) $$A=\left(V_{m}\times \rho_{w}\right)/{W^{\prime }}_{m}$$

(17) $$B=(V_{a}\times \rho_{w})/{W^{\prime }}_{m}$$

(18) $$E=e^{-{(H}_{1}-H_{L})K_{B}T}$$

where $V_{m}$ and $V_{a}$ describe the volume of adsorbed and absorbed water, respectively, $\rho_{w}$ is equal to the density of water, and ${W^{\prime }}_{m}$ is the mass of the dry sample. $k_{B}$ is Boltzmann’s constant and *T* is the absolute temperature.

## 3. Results

### 3.1. Thermogravimetric Analysis (TGA) of ROS

**Figure 2:**
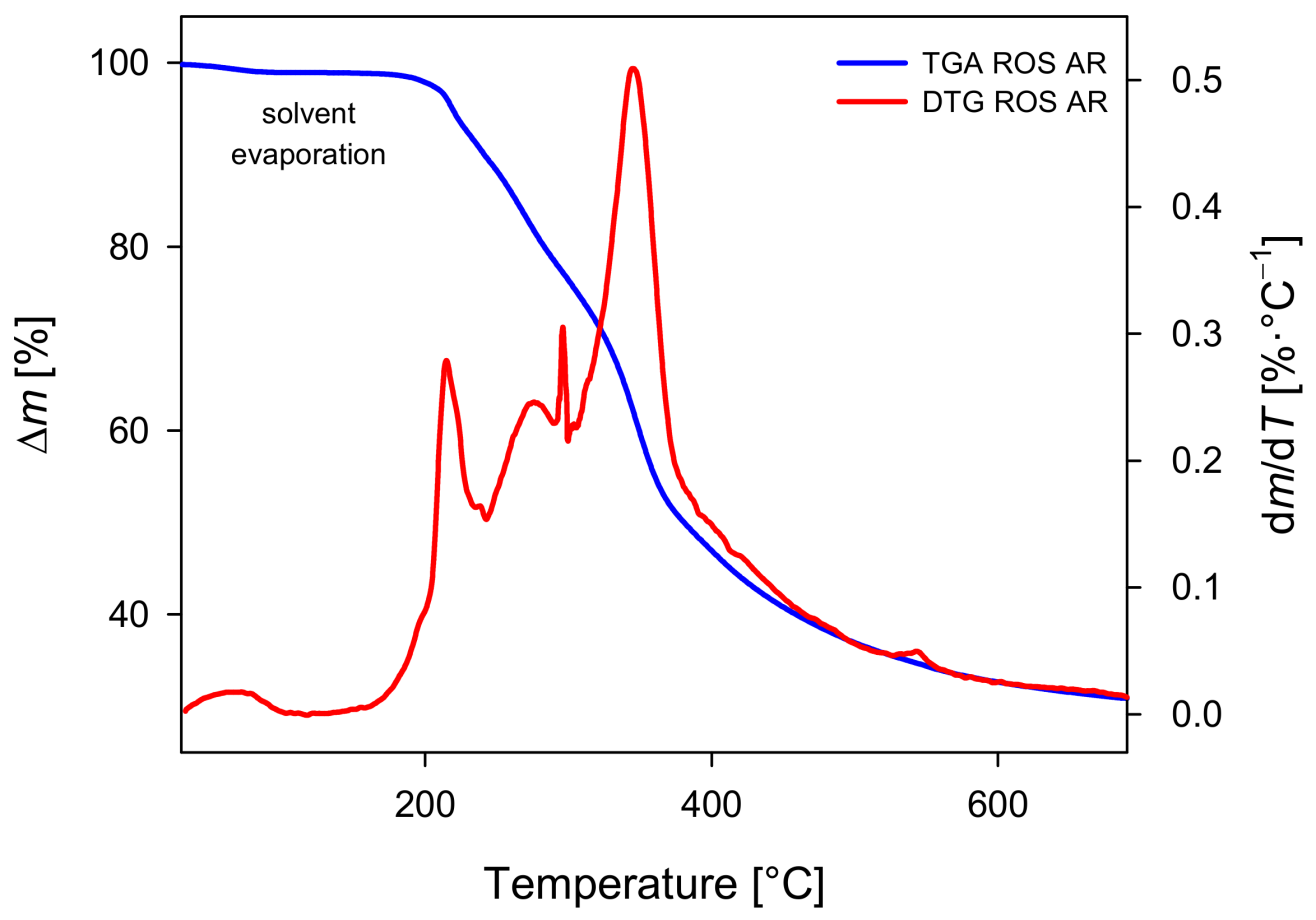
TGA and DTG of as-received rosuvastatin (ROS AR).

The thermal stability of as-received rosuvastatin (ROS AR) was characterised by TGA. The TGA curve of the rosuvastatin calcium sample (Figure 2) shows around 0.83% mass loss at around 30 and 100 °C, which is attributed to the evaporation of surface-adsorbed solvent. Between 140 and 170 °C, 0.1% of mass loss is observed, followed by a two-step thermal degradation process.

### 3.2. Differential Scanning Calorimetry (DSC) and Temperature-Modulated Differential Scanning Calorimetry (TMDSC) of ROS

**Figure 3:**
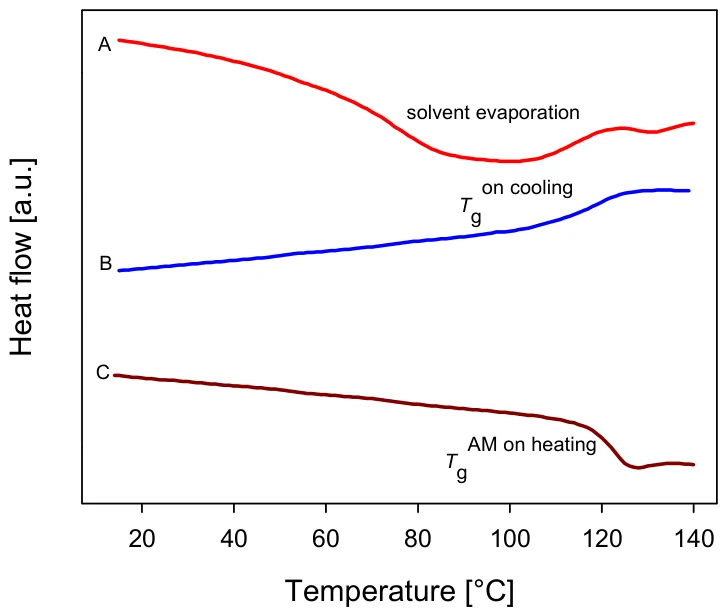
DSC curves of ROS showing (A) 1st heating (ROS AR; solvent evaporation), (B) cooling (glass transition of an amorphous material) and (C) 2nd heating (ROS AM; glass transition with an enthalpy relaxation of the amorphous material). All runs obtained at a 10 °C·min^−1^ heating rate.

Figure 3 shows the standard DSC traces of ROS AR at a temperature range from 15 to 140 °C with first heating at a 10 °C·min^−1^ heating rate, a cooling run and a second heating run. On the first heating curve there is one broad endothermic event, at around 40–120 °C with a peak maximum at 87 ± 2 °C, corresponding to a loss of surface solvent, and one small endothermic peak at around 130 °C (Figure 3, curve A). A similar observation has been reported by Belozerova et al. [52]. There is no sharp endothermic event arising from the melting transition, suggesting a lack of crystal network in the ROS AR sample.

On the cooling run one event is observed: a glass transition with *T*_g_^on cooling^ at 115.7 ± 0.6 °C with a change of heat capacity (Δ*c_p_*) equal to 0.38 ± 0.01 J·g^−1^ (Figure 3, curve B). On the 2nd heating curve, one event is observed (Figure 3, curve C): *T*_g_^on heating^ at 121.9 ± 0.3 °C with a Δ*c_p_* = 0.41 ± 0.01 J·g^−1^. The value of *T*_g_ on heating is significantly higher than on cooling; this is due to physical ageing of the material during cooling and heating below the glass transition temperature [53,54].

**Figure 4:**
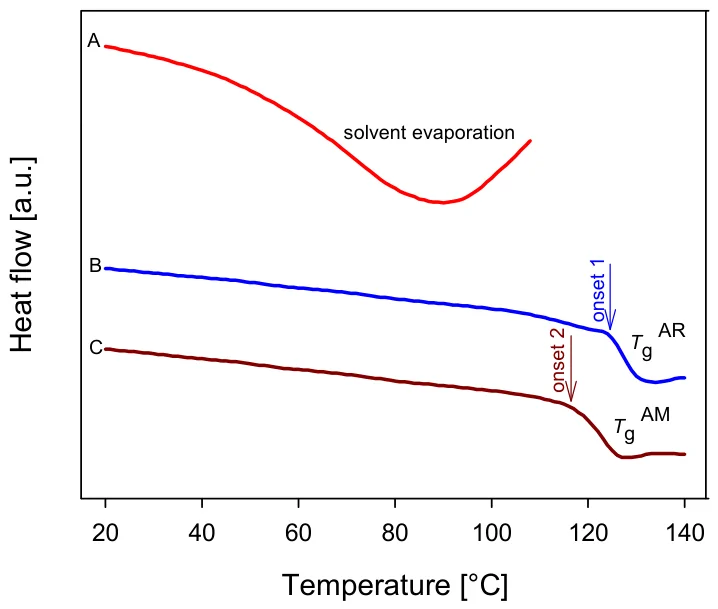
DSC curves of ROS AR (A) heating up to 110 °C in order to remove the solvent, (B) the heating of ROS AR after solvent removal (glass transition of AR sample) and (C) heating run ROS AM (glass transition of AM sample). All runs obtained at a 10 °C·min^−1^ heating rate.

To confirm the amorphous structure of ROS AR, a two-step experiment was performed (Figure 4). First, the sample was heated to 110 °C followed by cooling and reheating. On the second heating scan, the glass transition is clearly seen at 127.1 °C, confirming the amorphous nature of the material. Probably due to some structural ordering of the material due to physical ageing, the *T*_g_ is slightly higher than the freshly prepared amorphous form where *T*_g_ = 121.6 °C (ROS AM) (*relaxation polyamorphism* or *pseudopolyamorphism*) [28,32,55]. To confirm this speculation TMDSC was employed.

**Figure 5:**
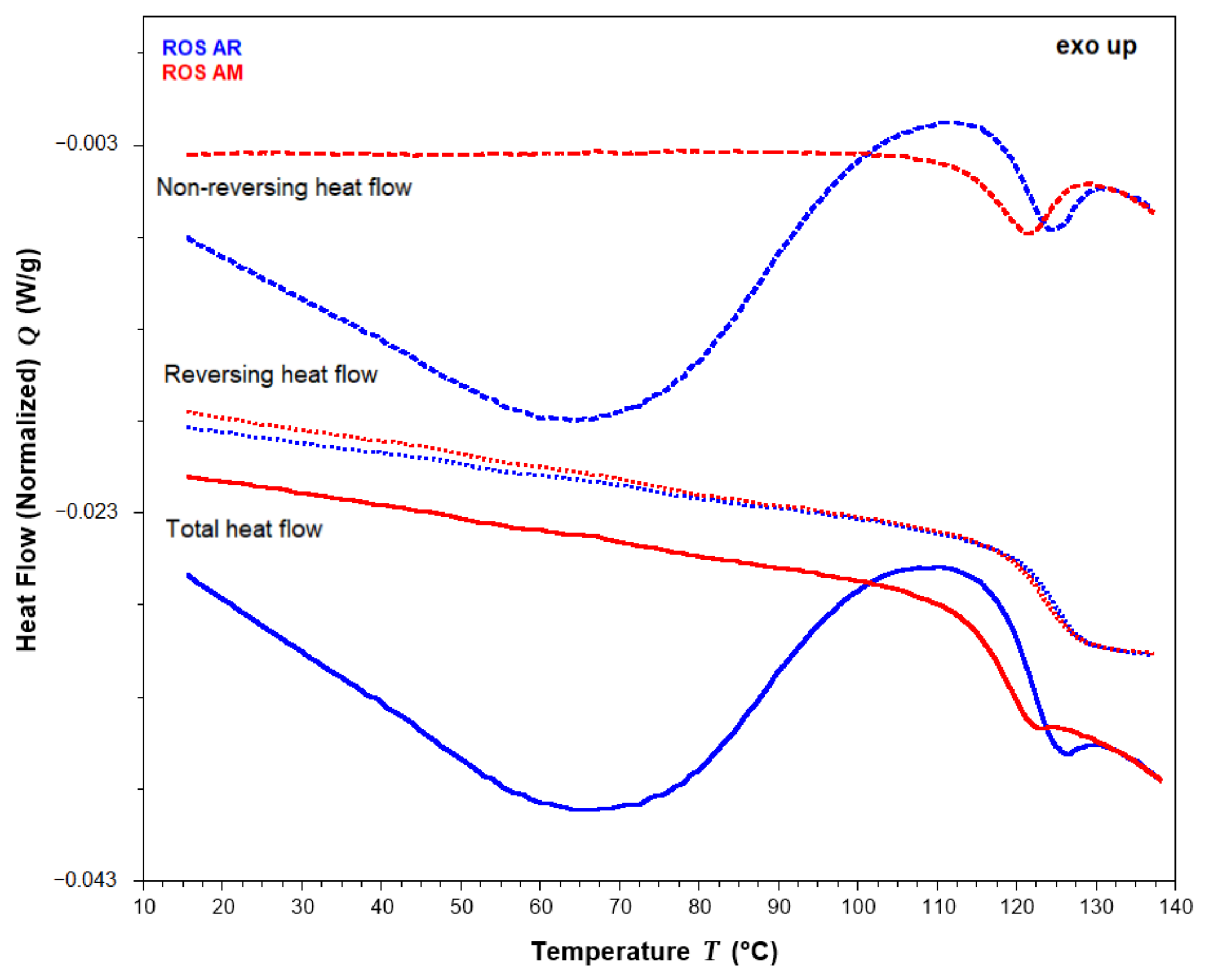
TMDSC curves of ROS AR (blue) and ROS AM (red) samples; solid—total heat flow, dotted—reversing heat flow, dashed—non-reversing heat flow. Curves obtained with an underlying heating rate 1 °C·min^−1^ and a temperature modulation with amplitude of 0.5 °C and period of 60 s from 10 to 140 °C.

By using TMDSC, the kinetic (solvent evaporation, enthalpy relaxation) and thermodynamic phenomena (glass transition) can be separated [56]. In Figure 5, TMDSC curves are shown for both rosuvastatin forms. The solvent evaporation and the enthalpy relaxation are clearly observed at the non-reversing (kinetic signal) and the glass transition at the reversing (thermodynamic signal) of the AR form. The curve of total heat flow of ROS AM shows glass transition overlapping with enthalpy relaxation. Notably, the differences observed between ROS AR and ROS AM originate primarily from the non-reversing signal associated with kinetic processes, whereas the glass transition observed in the reversing heat flow remains essentially unchanged. This indicates that the two forms differ mainly in their relaxation state and thermal history rather than in their glass transition behaviour, supporting the interpretation that ROS AR and ROS AM represent distinct amorphous states, consistent with a kinetic polyamorphic behaviour [28].

**Figure 6:**
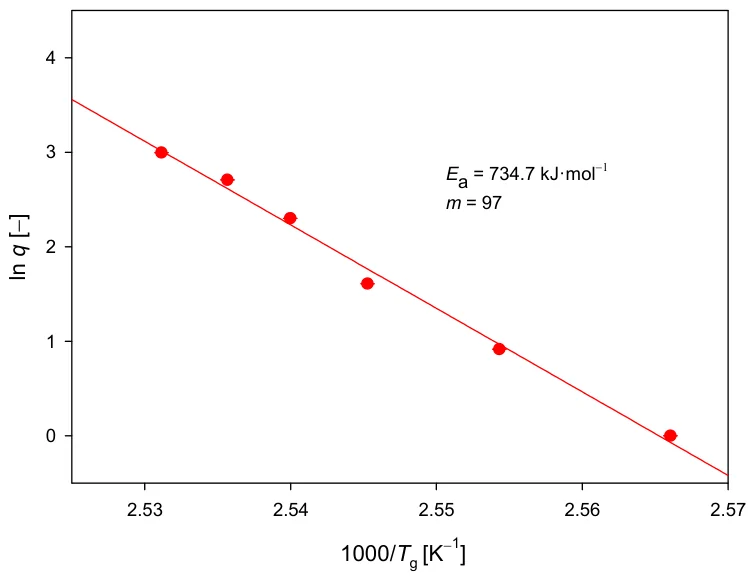
Determination of the activation energy (*E*_a_) and fragility parameter of rosuvastatin by DSC. “Arrhenius plot” of the logarithm of the heating rate, *q*, as a function of 1000/*T*_g_, where *T*_g_ is the temperature of the extrapolated onset of the glass transition step signal.

To address the molecular mobility and stability of amorphous rosuvastatin, its activation energy of the structural relaxation (*E*_a_(*T*_g_)) and fragility parameter (*m*) was calculated using the glass transition temperature dependency on heating rate (Table 1) [57]. *E*_a_ was estimated to be 734.7 kJ·mol^−1^ and *m* = 97 (Figure 6). Based on the classification system proposed by Angell [38], ROS can be classified as fragile glass. Other examples of APIs that create fragile glass are bifenazole (*E*_a_ = 402 kJ·mol^−1^ and *m* = 72) [58], carvedilol (*E*_a_ = 539 kJ·mol^−1^ and *m* = 91) [59], indapamide (*E*_a_ = 473 kJ·mol^−1^ and *m* = 66) [60] or lamotrigine (*E*_a_ = 655 kJ·mol^−1^ and *m* = 89) [58].

### 3.3. Powder X-Ray Diffractometry (PXRD) and Small-Angle X-Ray Scattering (SAXS) of ROS

**Figure 7:**
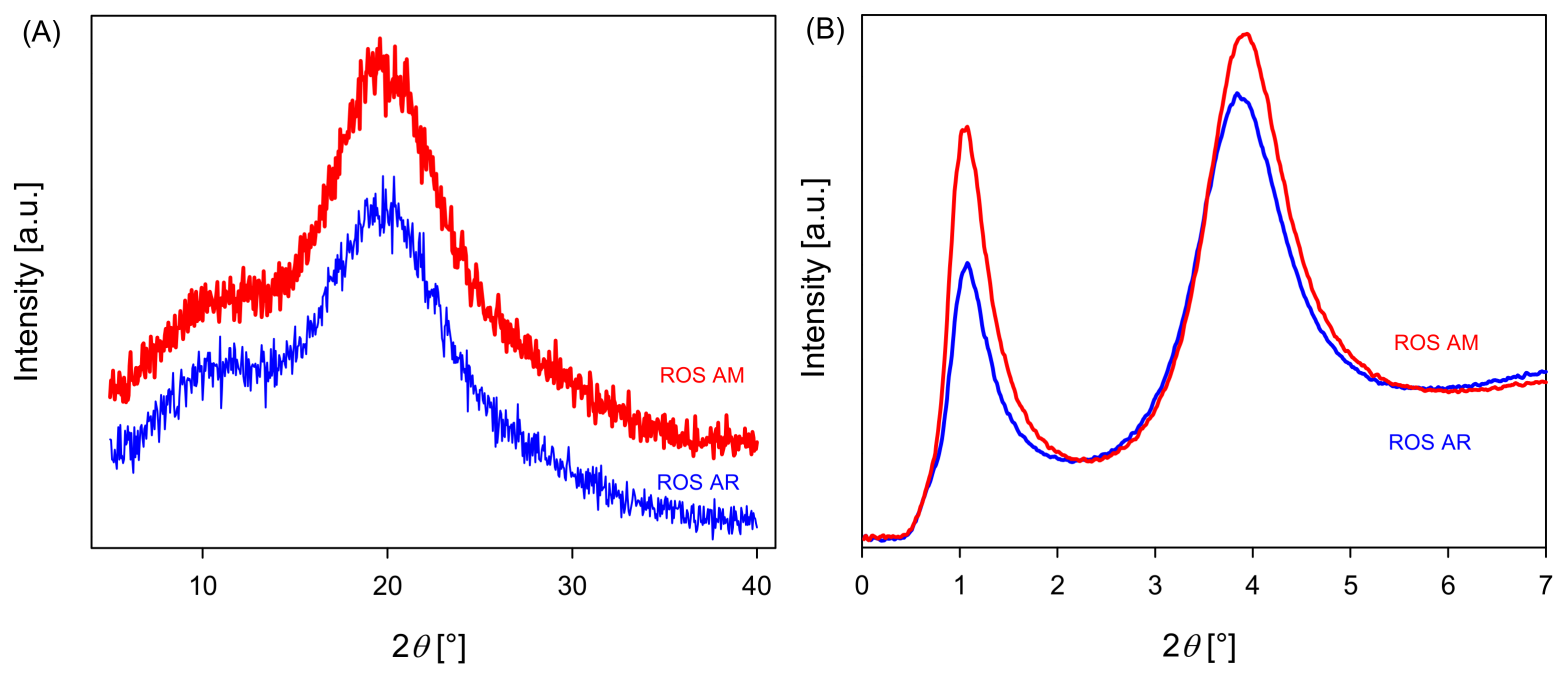
(**A**) PXRD analysis and (**B**) SAXS analysis of ROS AR and ROS AM.

To confirm the results obtained with DSC, powder X-ray diffractometry was conducted. PXRD analysis of ROS AR and ROS AM shows a “halo” that is characteristic of amorphous materials (Figure 7A).

A small-angle X-ray scattering experiment was performed to study the structure of ROS AR and ROS AM. Figure 7B shows two distinct broad peaks—the first appearing approximately at 1.2° 2θ, and the second at 4° 2*θ*. The presence of peaks suggests that the sample is partially ordered (short-range) in its structure with characteristic spacing, but their broadness indicates a degree of disorder or size distribution characteristic to amorphous solids [61]. Differences in the intensity of SAXS peaks may reflect variations in electron density fluctuations and structural heterogeneity within the amorphous phase [62,63]. The shift of the broad SAXS maxima towards higher scattering angles in ROS AM indicates a reduction in characteristic intermolecular distances and suggests a more densely packed amorphous structure compared with ROS AR [63]. This observation is consistent with the thermal analysis results and supports the hypothesis that the two materials differ in their degree of structural relaxation despite exhibiting the same amorphous character by PXRD and SSNMR.

### 3.4. Solid-State Nuclear Magnetic Resonance (SSNMR) of ROS

**Figure 8:**
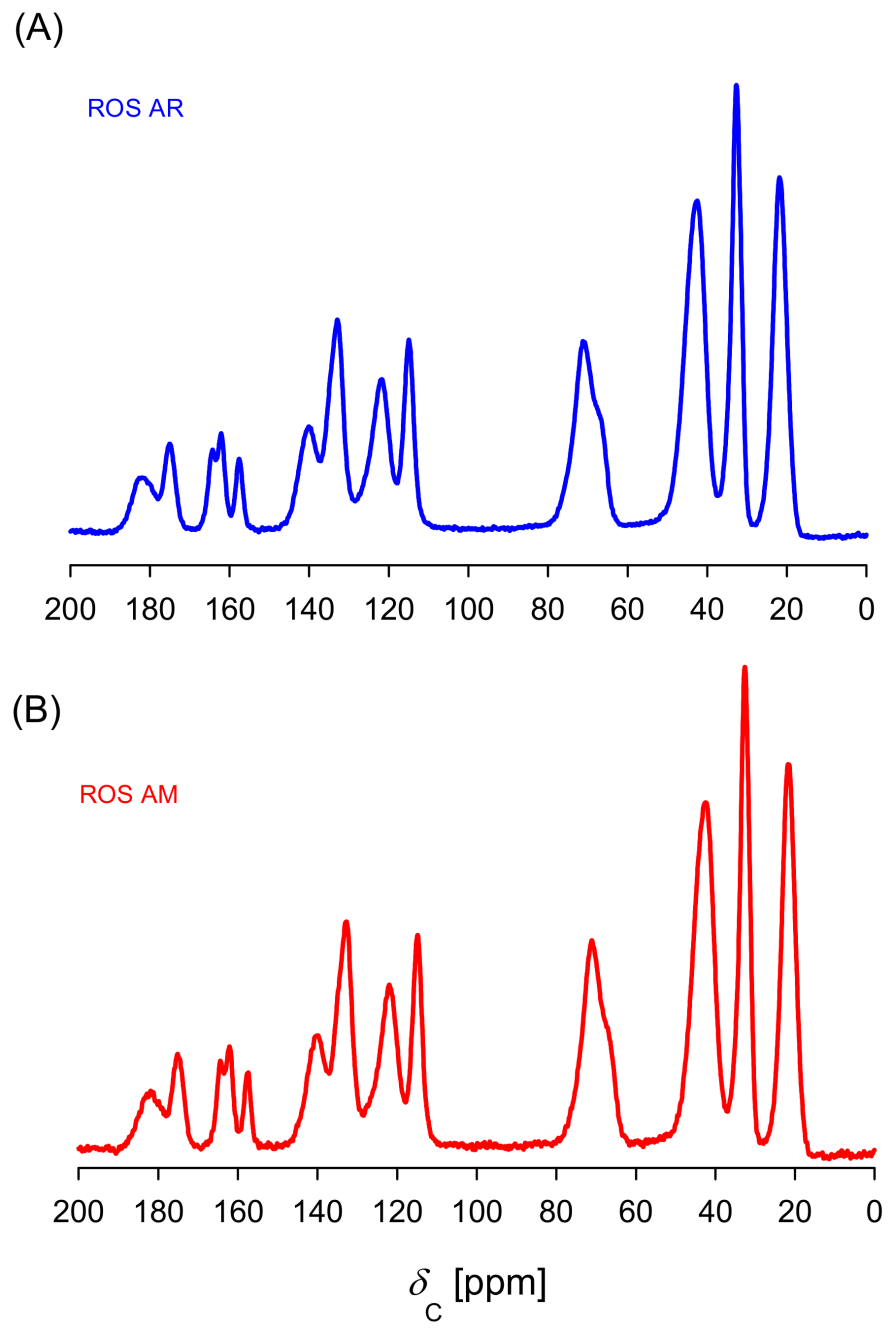
^13^C SSNMR analysis of (**A**) ROS AR and (**B**) ROS AM. Spectra recorded at 20 kHz spinning rate at 20 °C.

When comparing the ^13^C SSNMR of ROS AR (Figure 8A) and ROS AM (Figure 8B), no significant differences are observed, with broad peaks indicating that both forms are amorphous. Furthermore, both spectra were essentially identical, indicating a very similar molecular environment in both amorphous forms. Similar results for ROS AR were obtained by Belozerova et al. [52]. Additional ^19^F SSNMR measurements (Figure S2, Supplemntary Material) likewise revealed no detectable differences between the samples. These findings suggest that the structural differences detected by DSC, TMDSC, and SAXS are subtle and do not lead to discernible changes in the SSNMR spectra, supporting the interpretation that both materials represent closely related amorphous states differing primarily in their degree of structural relaxation.

### 3.5. Dynamic Vapour Sorption (DVS) of ROS

**Figure 9:**
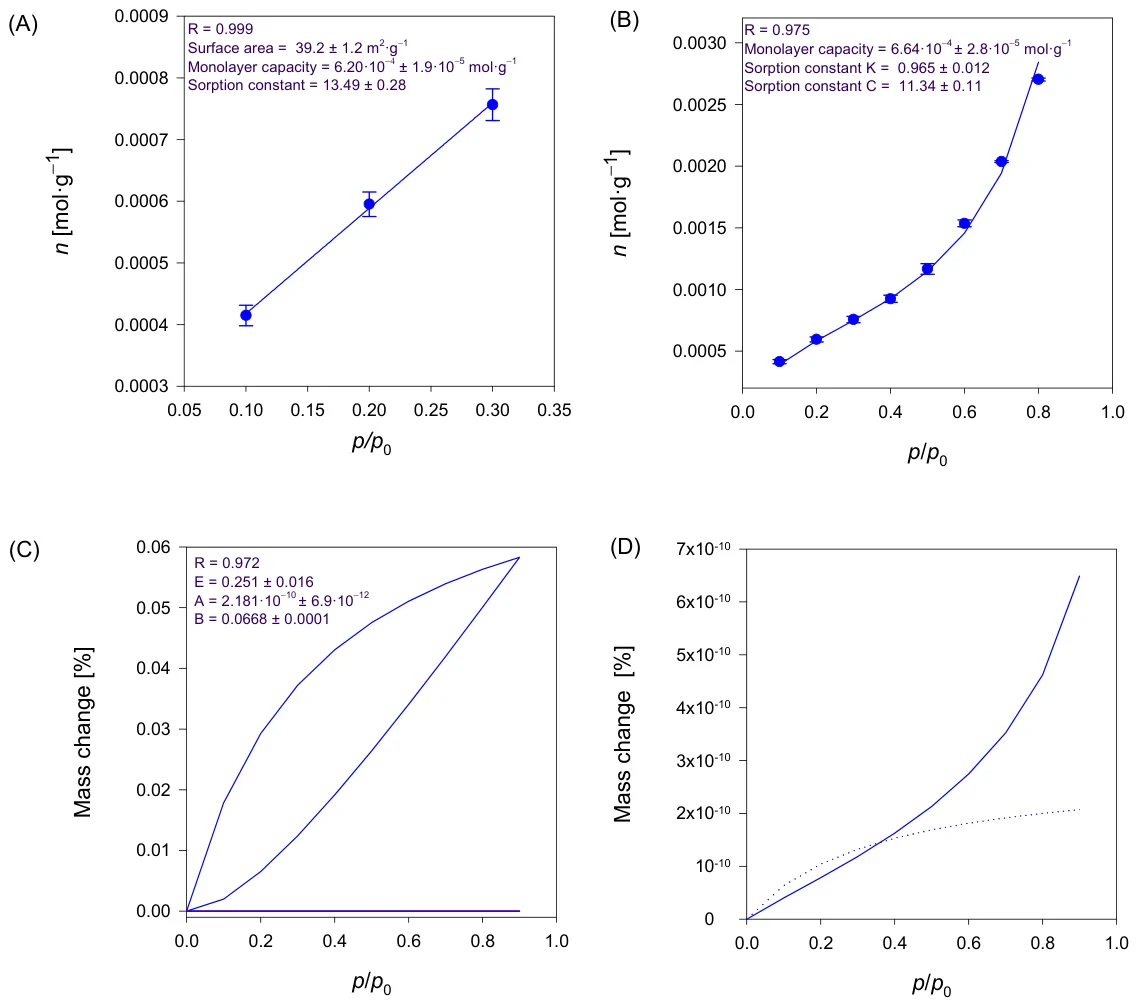
(**A**) BET analysis of ROS AR, (**B**) GAB analysis of ROS AR, (**C**) YN component plot for ROS AR, and (**D**) YN component plot for ROS AR, dotted—monolayer, solid—multilayer.

To determine the surface area of ROS AR, the Brunauer–Emmett–Teller (BET) equation was used [47]. The water adsorption isotherm is shown in Figure 9A. It exhibits a linear region between *p/p*_0_ = 0.10 and 0.30 and can be classified as sorption isotherm type II according to the IUPAC nomenclature [64]. This type of isotherm is given by a non-porous or macroporous adsorbent with unrestricted monolayer–multilayer adsorption [65]. The specific surface area measured by BET is 39.2 ± 1.2 m^2^·g^−1^ and can be considered as a moderate specific surface area compared to other results such as spray-dried raffinose stored in 0% RH, the specific surface area of which is 3.9 m^2^·g^−1^ [66], agarose composite aerogel—20.79 m^2^·g^−1^ [67] or nano-silica 100 m^2^·g^−1^ and 470 m^2^·g^−1^ [68]. The calculated monolayer capacity is 6.2·10^−4^ ± 1.9·10^−5^ mol·g^−1^, which indicates the small surface available for adsorption. Sorption constant *C* equals to 13.49 ± 0.28, which suggests a weak interaction between water molecules and rosuvastatin [47].

To further investigate sorption properties of rosuvastatin, the Guggenheim–Anderson–de Boer (GAB) model was used [48,49,50,69]. The monolayer capacity is similar to results obtained with the BET model and it is equal to 6.64·10^−4^ ± 2.8·10^−5^ mol·g^−1^, approximately. Sorption constant *C* equals to 11.34 ± 0.11, while sorption constant *K* describing the strength of the interaction between the absorbed multilayer is 0.965 ± 0.012. This means the water in ROS AR is bound in multilayers rather than only in a monolayer [70]. The GAB isotherm is shown in Figure 9B.

The Young and Nelson model (YN) was employed to determine if water is bound only to the surface of the rosuvastatin sample or is also bound in the bulk of the sample. Results suggest that the water is bound on the surface and in the bulk as well due to the visible bulk hysteresis present in Figure 9C [51]. This experiment also confirms that water adsorbs to the ROS AR in multilayers (Figure 9D).

Despite ROS AR’s ability to sorb water in both the surface and bulk regions, amorphous rosuvastatin remained remarkably resistant to recrystallisation as examined by PXRD (Figure S3, Supplementary Materials). This observation suggests that water uptake alone is insufficient to induce crystallisation under the investigated storage conditions.

Since ROS AR and ROS AM represent closely related amorphous states that differ mainly in the extent of short-range order, as previously demonstrated by DSC, TMDSC, PXRD, and SSNMR, similar moisture-induced phase behaviour would therefore be expected for ROS AM [71].

### 3.6. Characterisation of Carvedilol–Rosuvastatin Physical Mixtures, Their Co-Amorphous System (COAM CAR-ROS), and Stability Studies of COAM CAR-ROS System

Following the characterisation of rosuvastatin, its co-amorphous systems with carvedilol were examined to describe thermal behaviour and assess its suitability as a potential FDC formulation.

Identification of potential interactions between APIs in FDC is of paramount importance at an early stage of the formulation development process [72]. Thermal analysis is frequently used to evaluate the drug–drug and drug–excipient compatibility [73,74]. In this study, TGA and DSC were employed for this purpose.

**Figure 10:**
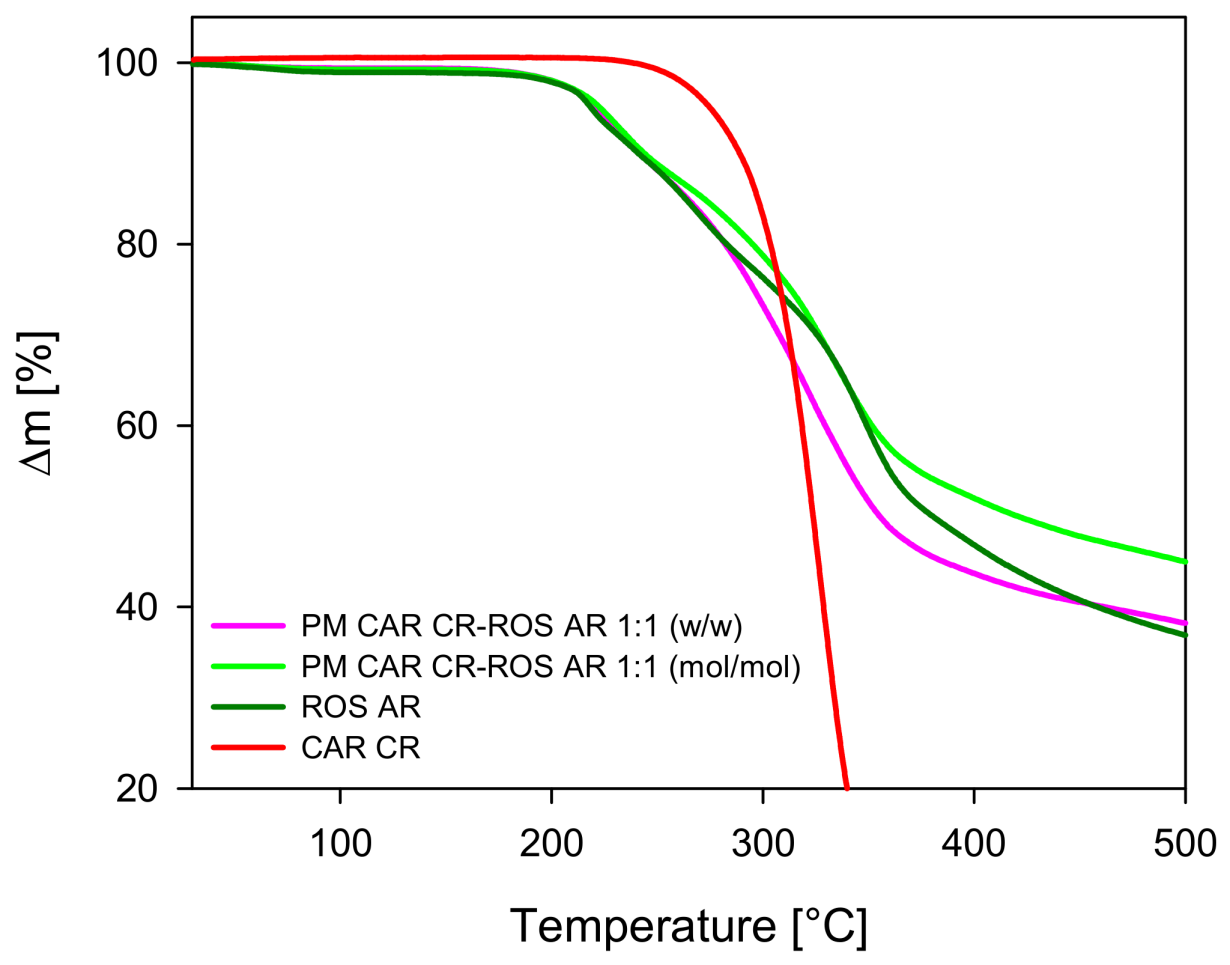
TG analysis of ROS AR, CAR CR, their 50:50 (*w*/*w*) physical mixture (PM CAR CR-ROS AR 1:1 (*w*/*w*) and their 1:1 (mol/mol) physical mixture (PM CAR AM-ROS AM 1:1 (mol/mol). Curves were obtained at a 10 °C·min^−1^ heating rate.

CAR begins to lose mass at around 250 °C (Figure 10) due to chemical decomposition, which is consistent with previous reports [59].

A 1:1 (*w*/*w*) physical mixture of the two compounds experiences thermal decomposition at around 246 °C, whereas the 1:1 (mol/mol) physical mixture decomposes at around 259 °C (Figure 10).

TGA curves of the individual drugs and the 50:50 (*w*/*w*) CAR CR-ROS AR physical mixture indicate that no degradation due to the interaction between the drugs takes place and that the ingredients change individually in the mixture, thereby suggesting no chemical incompatibility.

Standard DSC traces of carvedilol at a temperature range from 5 to 140 °C with a 10 °C·min^−1^ heating rate, a cooling run and a second heating are shown in Figure S1 (Supplementary Material). On the first heating (Figure S1, curve A), the DSC curve shows one sharp endothermic peak with an onset at *T*_fus_ = 113.9 ± 0.1 °C and an enthalpy of fusion Δ_fus_*h* = 117.7 ± 0.9 J·g^−1^ due to melting. During the cooling run (Figure S1, curve B) from the molten state, one event is observed: a glass transition with T_g_^on cooling^ at 35.0 ± 0.3 °C and change of heat capacity (Δ*c_p_*) of 0.79 ± 0.02 J·g^−1^. One event is observed on the second heating run (Figure S1, curve C): T_g_^on heating^ at 38.0 ± 0.1 °C with Δ*c_p_* = 0.82 ± 0.02 J·g^−1^ overlapped with an enthalpy recovery peak. Similarly to the ROS AM, the value of *T*_g_ on heating is higher than on cooling due to physical ageing of the material. The obtained data were consistent with the previously reported results [59].

**Figure 11:**
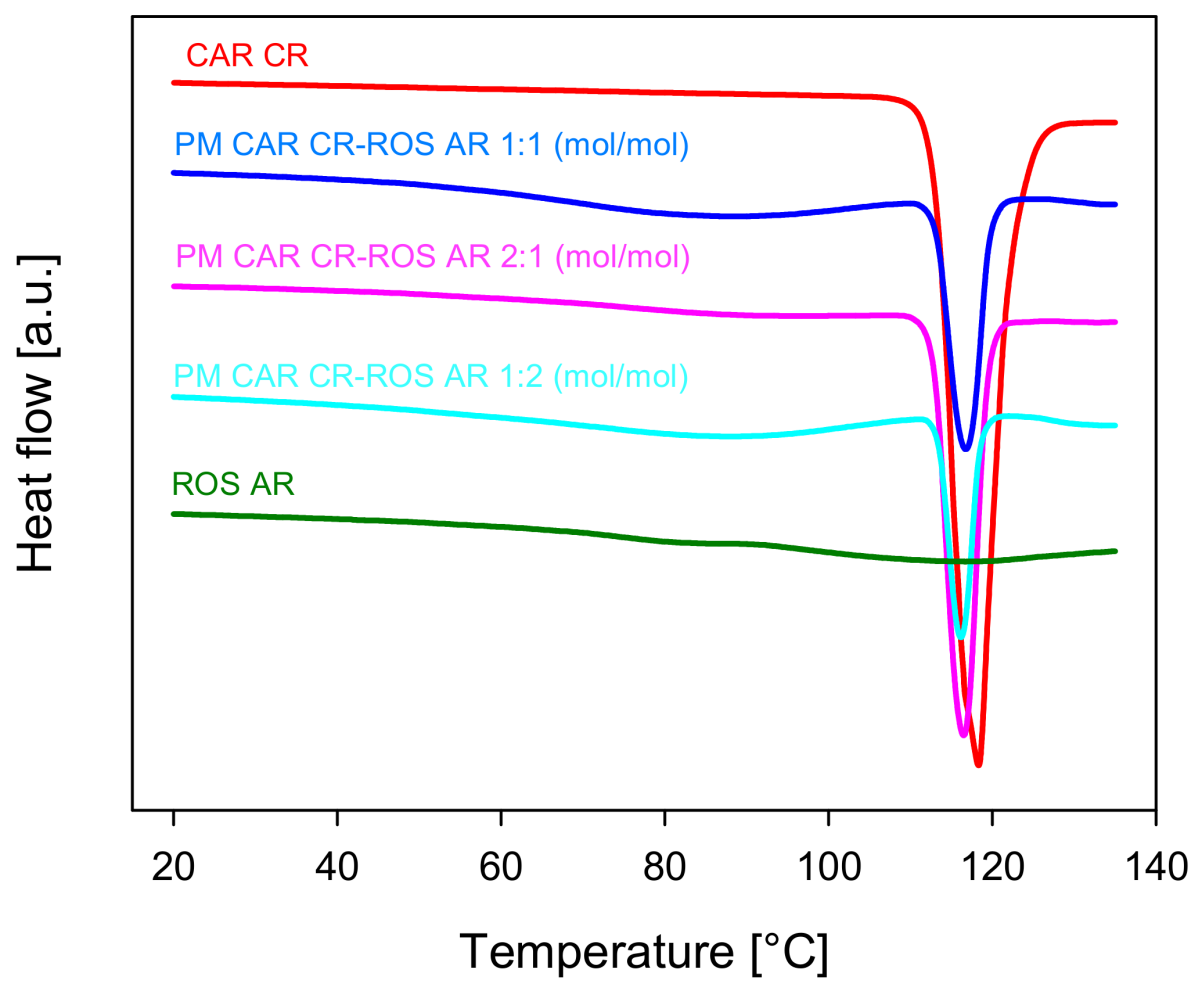
DSC curves of the 1:1, 2:1, 1:2 mol/mol physical mixtures of carvedilol and rosuvastatin (PM CAR CR-ROS AR 1:1 (mol/mol), PM CAR CR-ROS AR 2:1 (mol/mol) and PM CAR CR-ROS AR 1:2 (mol/mol)), CAR CR, ROS AR—1st heating runs. Data obtained at a 10 °C·min^−1^ heating rate.

The DSC curves of the 2:1, 1:1, and 1:2 (mol/mol) physical mixtures of carvedilol and rosuvastatin show the expected sharp carvedilol melting peak (Figure 11). The onset of *T*_m_ is close to the standalone crystalline carvedilol, and the total enthalpy of this peak is estimated to be around 50, 28, and 18 J·g^−1^, respectively, which is similar to the weight fraction enthalpy of the individual components. Table 2 suggests no physical or chemical interaction between the components.

**Figure 12:**
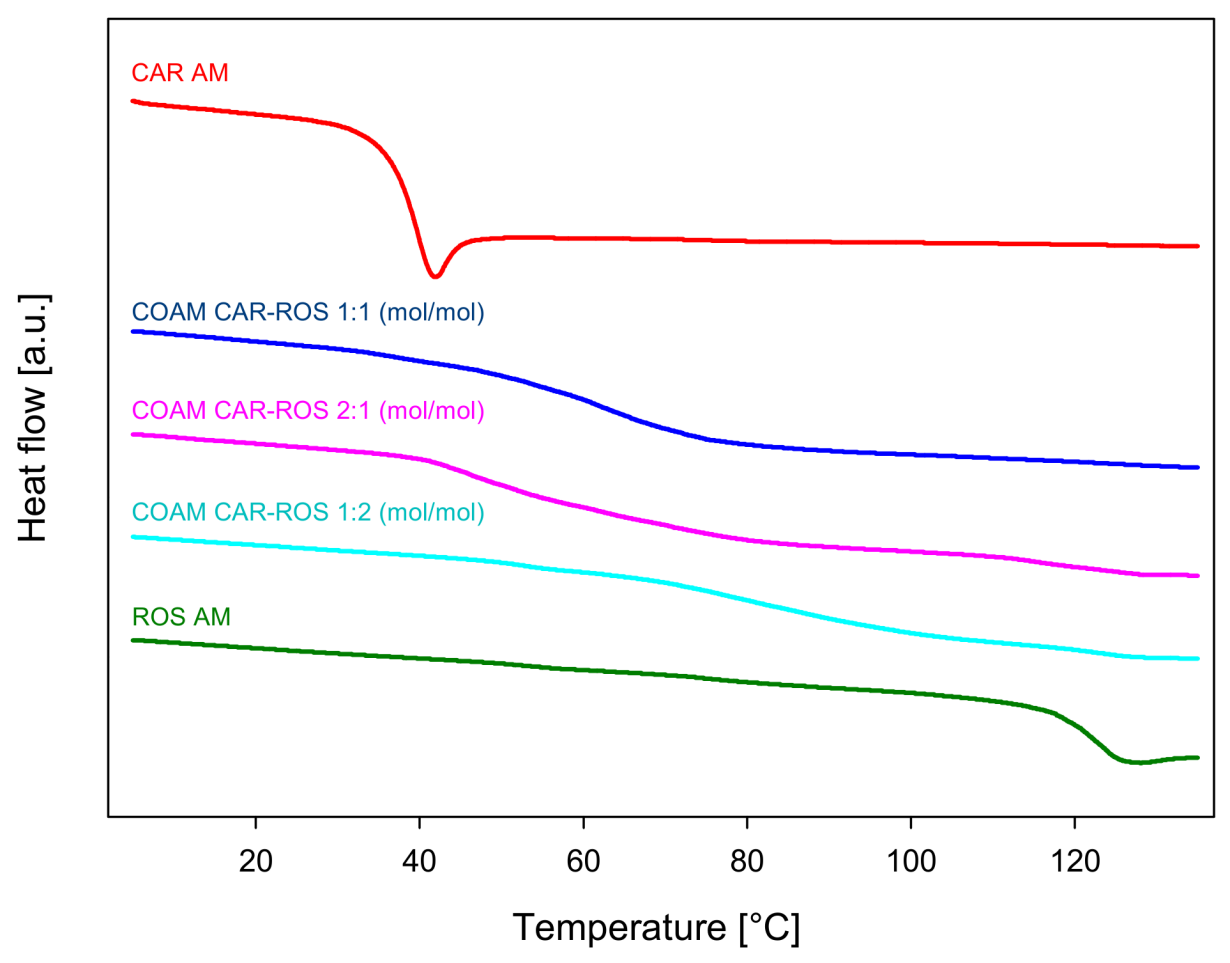
DSC curves of the 1:1, 2:1, 1:2 mol/mol co-amorphous system of carvedilol and rosuvastatin (COAM CAR-ROS 1:1 (mol/mol), COAM CAR-ROS 2:1 (mol/mol) and COAM CAR-ROS (1:2 mol/mol)), CAR CR, ROS AR—2nd heating runs. Data obtained at a 10 °C·min^−1^ heating rate.

In the second DSC heating run, both components are amorphous (Figure 12). Neither melting nor cold crystallisation is observed for co-melted physical mixtures; only a single glass transition is observed. In contrast to the physical mixtures, the co-amorphous formulations exhibit a single glass transition, indicating that homogeneous molecular mixing is achieved after the co-melting process. Compounds that are not miscible would show two separate glass transitions, one for each compound, while the co-melted CAR-ROS mixture only shows a single glass transition at a temperature intermediate between the *T*_g_ of pure drugs, with the temperature depending on the composition.

The efficiency of mixing both components can be assessed by the Couchman–Karasz (CK) equation [46]. In the glassy single-phase systems, the *T*_g_ of the amorphous multi-component material is usually found between the glass transitions of the individual components. Mixing a drug with another molecule (another drug or excipient) with a high *T*_g_ can improve physical stability due to the increased *T*_g_ of the mixture (anti-plasticization effect) [16].

Strong negative deviations from the predicted *T*_g_ values were observed for all systems (Table 3). This might be an indication of non-ideal mixing behaviour and/or a possibility of specific interactions occurring between the APIs [75], an increase in free volume [76] or changes in molecular packing [77]. The progressive increase in *T*_g_ with increasing ROS content demonstrates the anti-plasticising effect of rosuvastatin. The high *T*_g_ of ROS elevates the glass transition temperature of the co-amorphous systems, which is expected to decrease molecular mobility and improve resistance to recrystallisation during storage.

**Figure 13:**
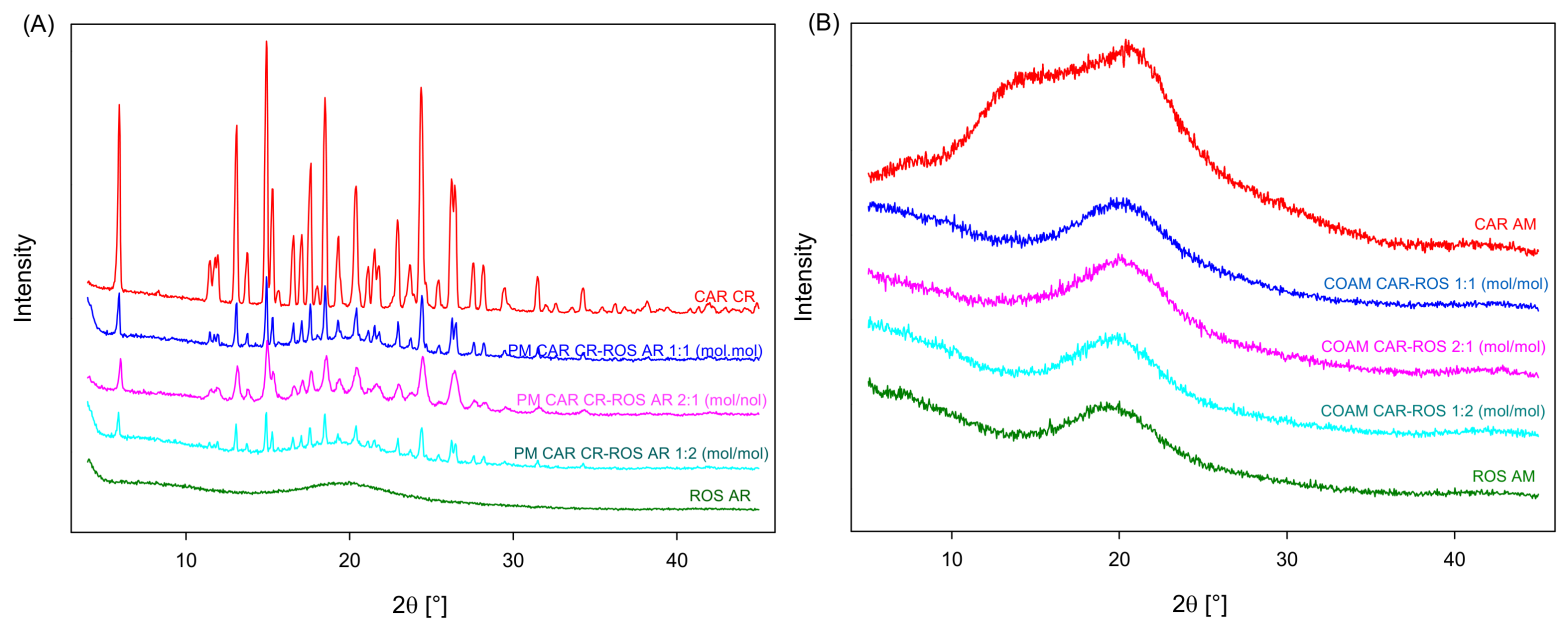
PXRD analysis of (**A**) CAR CR, PM CAR CR-ROS AR 1:1 (mol/mol), PM CAR CR-ROS AR 1:2 (mol/mol), PM CAR CR- ROS AR 2:1 (mol/mol)), and ROS AR and (**B**) CAR AM, COAM CAR-ROS 1:1 (mol/mol), COAM CAR-ROS 1:2 (mol/mol), COAM CAR-ROS 2:1 (mol/mol)) and ROS AM).

To evaluate whether CAR and ROS formed fully amorphous systems, PXRD analysis was conducted. Diffractograms showing patterns of crystalline and amorphous components, their physical mixtures and co-amorphous systems in various molar ratios are shown in Figure 13A,B. Results indicate that CAR and ROS form co-amorphous systems in 1:1, 1:2 and 2:1 molar ratios, considering the “halos” visible in Figure 13B.

**Figure 14:**
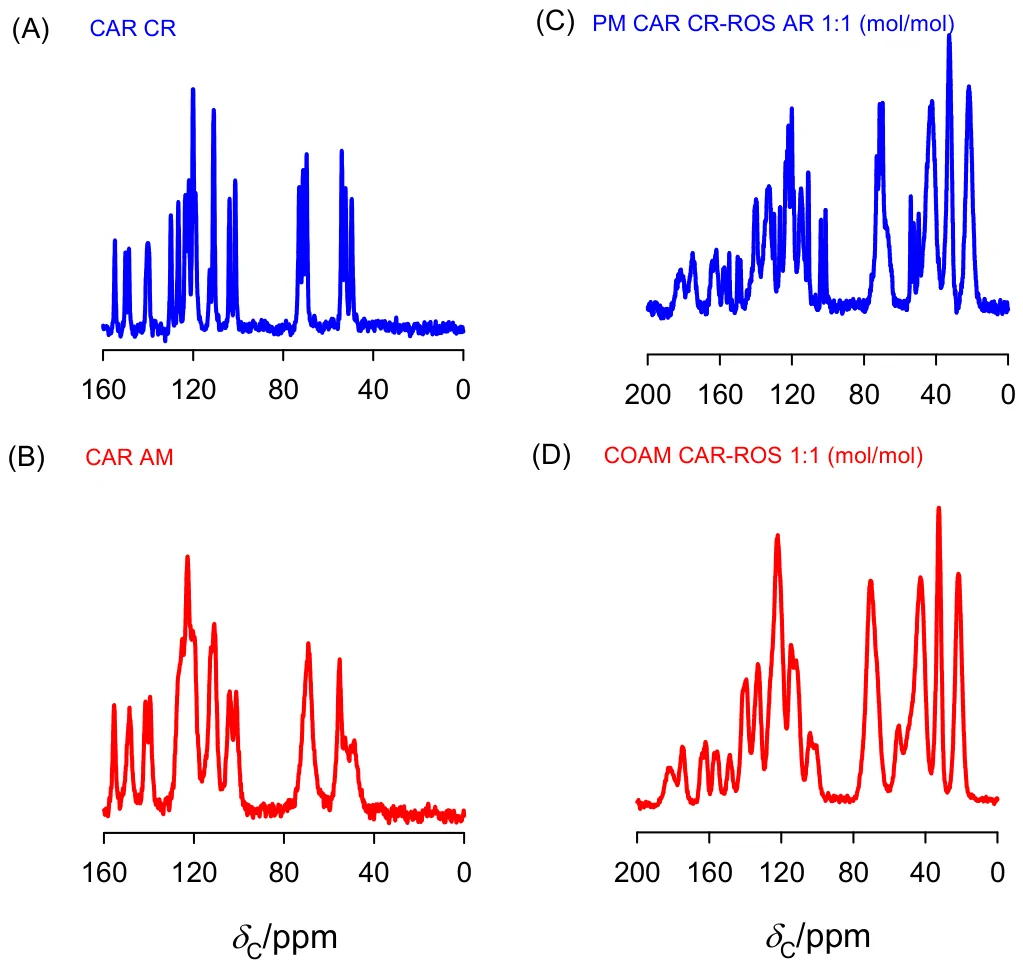
^13^C SSNMR analysis of (**A**) CAR CR, (**B**) CAR AM, (**C**) PM CAR CR-ROS AR 1:1 (mol/mol), and (**D**) COAM CAR-ROS 1:1 (mol/mol). Spectra recorded at 20 kHz spinning rate at 20 °C.

For the spectra of ^13^C SSNMR, the peaks associated with the amorphous form of CAR are broader than those related to the crystalline material (Figure 14A and B respectively). Additionally, the spectral data shows that crystalline carvedilol was in its polymorphic form II [78,79]. The spectroscopic analysis further confirmed the results obtained by DSC and PXRD.

The ^13^C SSNMR spectrum of the COAM CAR–ROS 1:1 system consisted of broadened resonances characteristic of an amorphous phase compared to the 1:1 molar ratio physical mixture of its components (Figure 14C) and contained signals attributable to both CAR and ROS (Figure 14D). No significant chemical-shift changes relative to the corresponding amorphous APIs were observed, suggesting that if any intermolecular interactions are present, they are either weak or insufficient to induce detectable changes in the standard ^13^C SSNMR spectra. Nevertheless, the absence of spectral changes does not preclude molecular-level mixing, which is strongly supported by the single glass transition observed by DSC.

**Figure 15:**
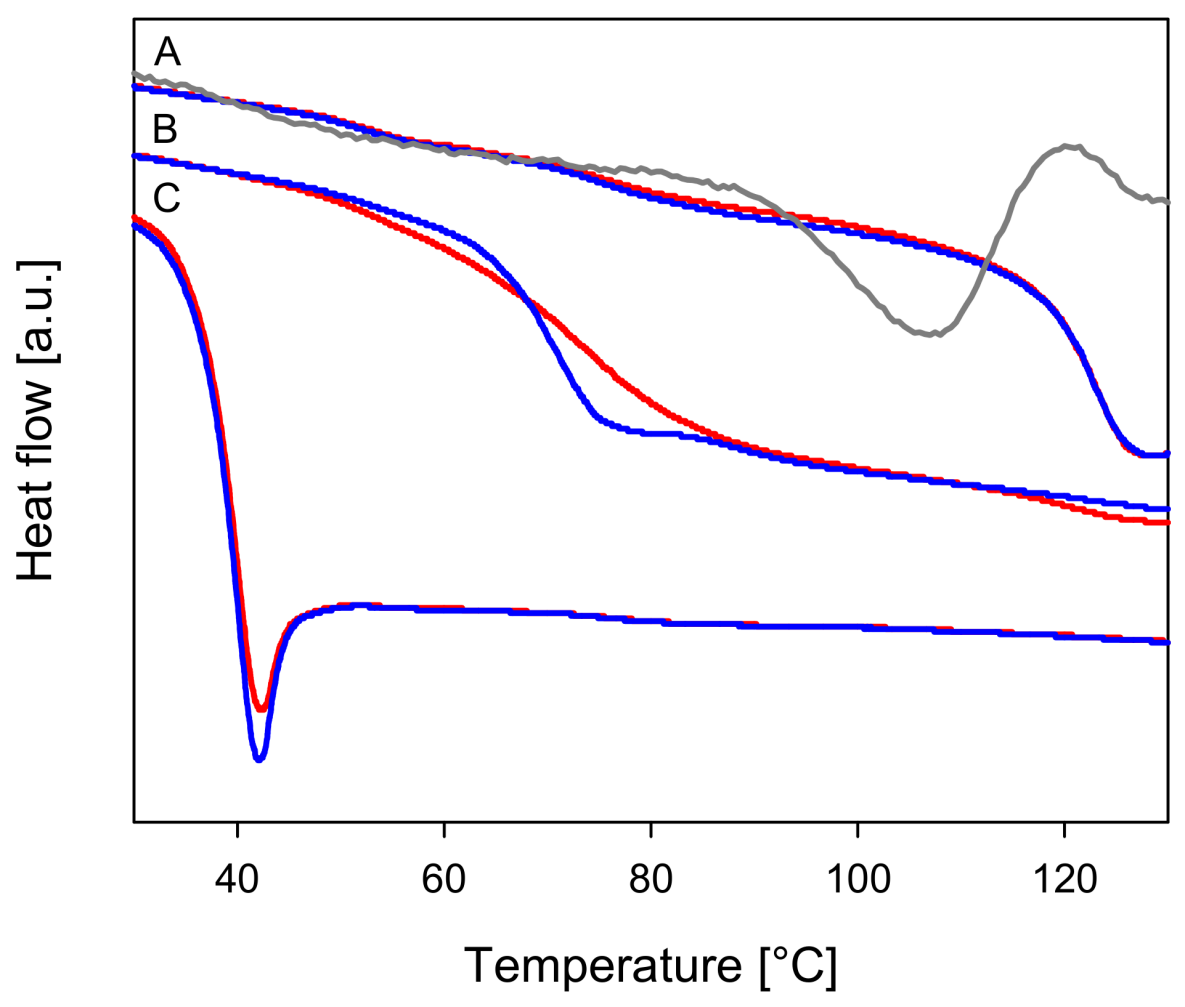
DSC curves of obtained amorphous samples (red) and stored in 45 °C for 1440 min in DSC (blue) and for three years in incubator (grey) for (A) ROS AM, (B) COAM CAR-ROS 1:1 (mol/mol), and (C) CAR AM. Curves were obtained at a 10 °C·min^−1^ heating rate.

Physical ageing studies were performed to evaluate the stability of the obtained systems. The enthalpy of relaxation showed only a negligible change with a storage time at 45 °C for CAR AM (Figure 15, curve blue C). Since the storage temperature was above the *T*_g_ of amorphous carvedilol, the observed difference is due to the experimental uncertainty of the measurement. Furthermore, no evidence of crystallisation was detected in the stored CAR sample. In contrast, a noticeable increase in the enthalpy of relaxation was observed for COAM CAR-ROS 1:1 (mol/mol) (Figure 15, curve blue B) indicating progressive structural relaxation during storage. Notably, ROS AM exhibited no changes after being stored at 45 °C for 1440 min (Figure 15, curve blue A). However, a marked increase in the enthalpy of relaxation clearly increased after three years of storage at 45 °C (Figure 15, curve grey A). The long-term storage results demonstrate that amorphous rosuvastatin remains thermodynamically driven towards structural equilibration over timescales of years; however, this relaxation process occurs without detectable crystallisation, highlighting the exceptional resistance of the material to devitrification.

**Figure 16:**
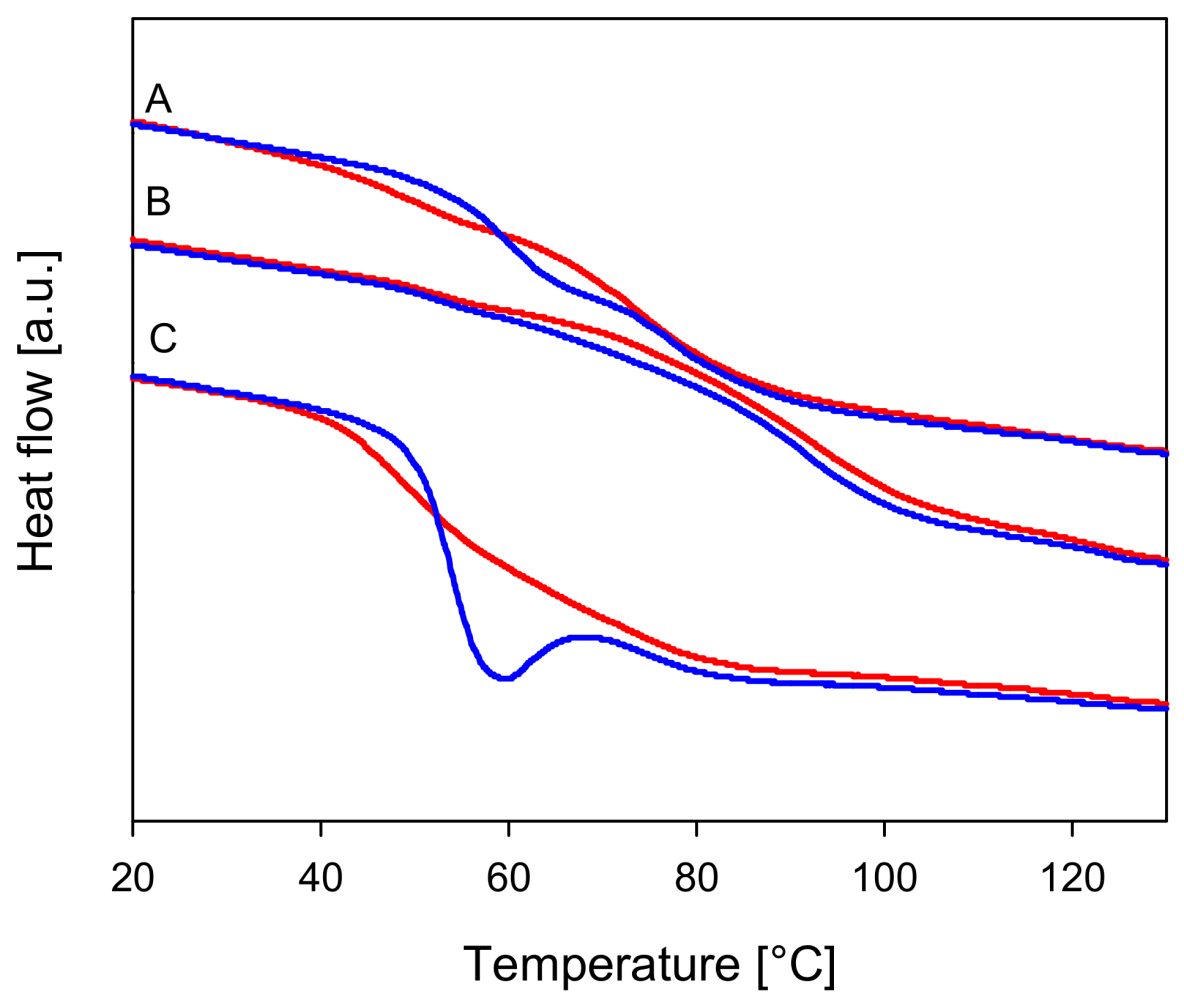
DSC curves of obtained amorphous samples (red) and stored in 30 °C for 1440 min (blue) for (A) COAM CAR-ROS 1:1 (mol/mol), (B) COAM CAR-ROS 1:2 (mol/mol), and (C) COAM CAR-ROS 2:1 (mol/mol). Curves were obtained at a 10 °C·min^−1^ heating rate.

To compare the ageing behaviour of ROS-CAR co-amorphous system with various molar ratio, samples were stored at 30 °C (Figure 16). After 1440 min of ageing, the DSC curve of COAM CAR-ROS 1:2 (mol/mol) exhibited only a slight shift which can be attributed to enhanced stabilisation of the system by higher ROS content (Figure 16, curve blue B). COAM CAR-ROS 2:1 (mol/mol) showed the greatest increase in enthalpy of relaxation, likely due to its relatively low *T*_g_ (Figure 16, curve blue C).

COAM CAR-ROS 1:1 (mol/mol) was chosen for further investigation of the physical stability and ageing behaviour. Co-amorphous systems with components in a 1 to 1 molar ratio are frequently reported to exhibit enhanced physical stability, which has been attributed to the possibility of optimal intermolecular interaction and formation of drug–drug heterodimers between two components [12,80]. For example, the co-amorphous system of CAR and *L*-aspartic acid (ASP) [73], as well as naproxen with cimetidine [80], have been shown to exhibit maximum stability at equimolar composition.

In contrast, an excess of either component has been reported to reduce physical stability and may promote recrystallisation, most likely due to the formation of homodimer aggregates that can act as nucleation sites. In the CAR-ASP system, non-equimolar compositions containing excess CAR or ASP exhibited faster recrystallisation compared to a 1:1 molar system [81].

**Figure 17:**
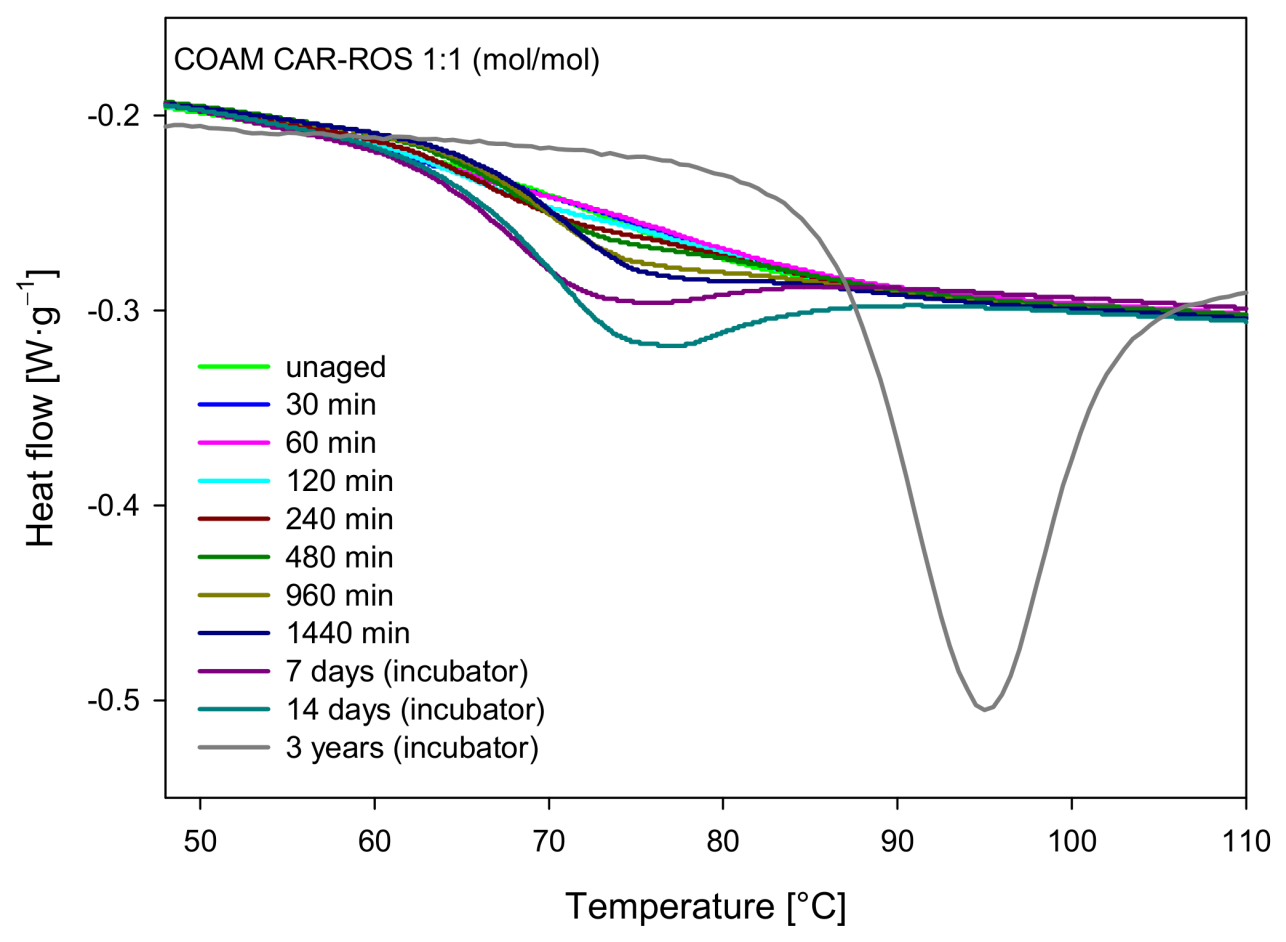
DSC curves of co-amorphous carvedilol–rosuvastatin system 1:1 (mol/mol) stored at 45 °C for 30, 60, 120, 240, 480, 960 and 1440 min in the DSC and 7, 14 days and 3 years in an incubator. Curves were obtained at a 10 °C·min^−1^ heating rate.

DSC analysis of samples stored in the incubator for over three years showed increasing enthalpy of relaxation and no signs of recrystallisation (Figure 17). The pronounced ageing observed at 45 °C compared to 30 °C agrees with the established temperature dependence of relaxation kinetics, where increased molecular mobility closer to *T*_g_ accelerates structural relaxation [81,82]. The observed long-term stability of the CAR–ROS system may therefore arise not only from molecular-level mixing but also from the anti-plasticising effect of rosuvastatin, which reduces molecular mobility by increasing the *T*_g_ of the co-amorphous phase.

**Figure 18:**
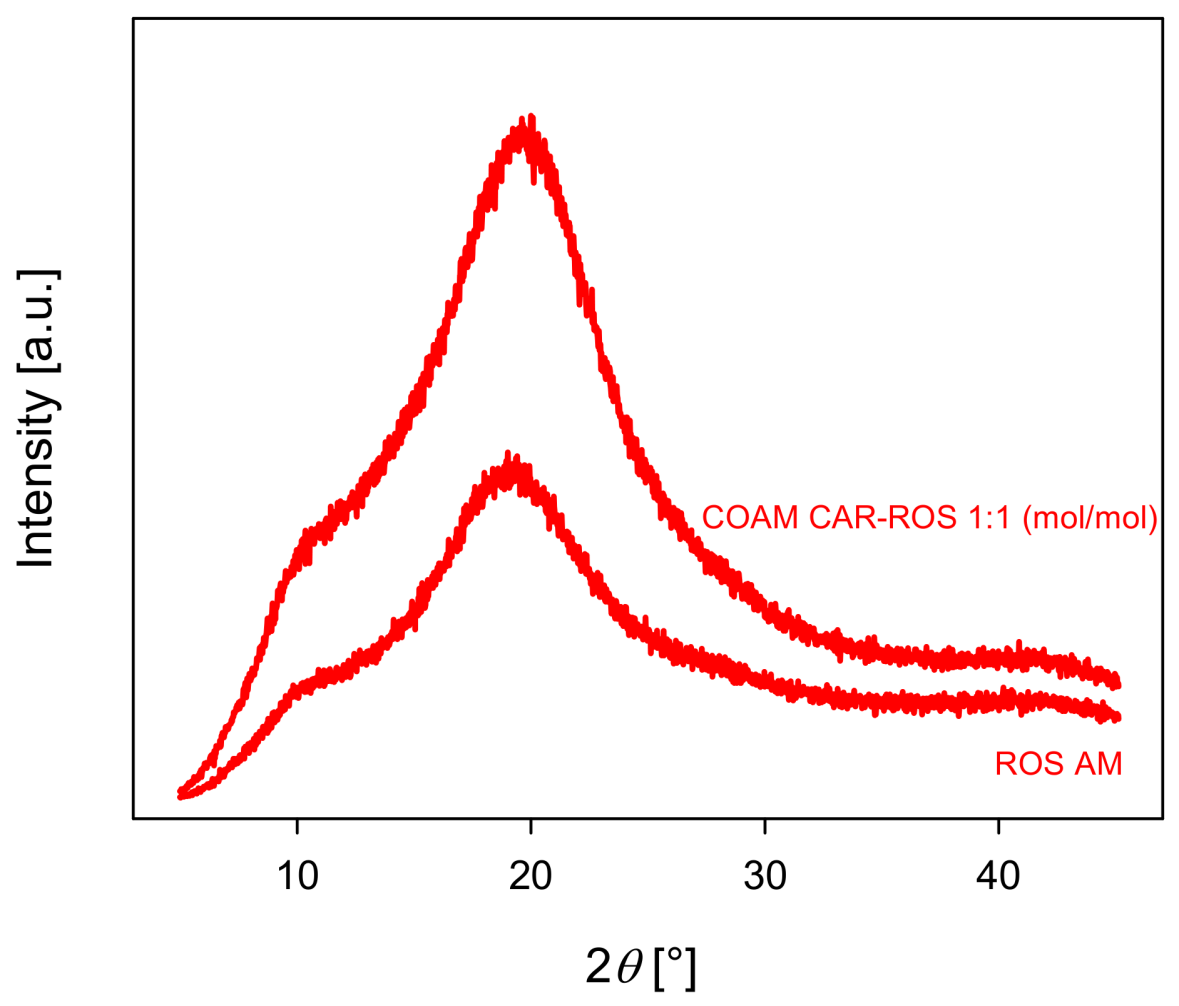
PXRD of ROS AM and COAM CAR-ROS 1:1 (mol/mol) after three years of storage at 45 °C.

PXRD analysis of samples of ROS AM and COAM CAR-ROS 1:1 (mol/mol) stored for over three years at *T*_a_ = 45 °C showed no signs of recrystallisation (Figure 18), which confirmed the results obtained by DSC. However, the stability of the co-amorphous system is formulation specific and may vary with composition. Therefore, the conclusion regarding long-term stability should not be directly extrapolated to non-equimolar CAR-ROS COAMs.

## 4. Discussion

The present study provides a comprehensive evaluation of the thermal behaviour, solid-state properties, and stability of rosuvastatin calcium (ROS) and its co-amorphous systems with carvedilol (CAR), offering insights relevant to the development of fixed-dose combination formulations. The TGA and DSC results confirm that ROS as received (ROS AR) is an amorphous material with residual solvent, as indicated by the initial mass loss and the broad endothermic event observed during the first DSC heating cycle. The absence of a melting endotherm indicates the lack of long-range crystalline order (amorphous character of the material). These findings are consistent with previous reports describing the solid state of rosuvastatin [24,52].

The glass transition behaviour of ROS AR and ROS AM, investigated by DSC and TMDSC, demonstrates that both forms exhibit similar *T*_g_ values, with differences attributed to physical ageing. PXRD, SAXS, and ^13^C SSNMR analyses corroborate the amorphous nature of both forms, with SAXS revealing subtle structural ordering (short-range) that does not translate into crystallinity. The combined DSC, TMDSC and SAXS results suggest that ROS AR and ROS AM represent distinct amorphous states differing in their degree of structural relaxation. Such behaviour is consistent with the concept of kinetically generated polyamorphic states, where glasses of identical chemical composition exhibit different structural organisation and relaxation behaviour as a consequence of different preparation pathways and thermal histories [28,29,30,34,35,36,37].

Water sorption analysis of ROS AR revealed a moderate specific surface area and multilayer adsorption behaviour. The Young–Nelson model confirmed that water is bound on the surface and in the bulk as well due to the visible bulk hysteresis, suggesting that moisture uptake may influence physical stability under humid conditions [83]. However, the low monolayer capacity and weak sorption constants suggest limited risk of moisture-induced recrystallisation, which was further confirmed by PXRD analysis showing no evidence of crystallisation after the DVS experiments (Figure S3, Supplementary Materials).

The formation of co-amorphous CAR–ROS systems was confirmed by DSC and PXRD. The presence of a single *T*_g_ in all compositions indicates miscibility at the molecular level. Negative deviations from the Couchman–Karasz equation suggest non-ideal mixing, likely due to denser packing, changes in free volume or loss in the number and strength of hydrogen bonding upon mixing two components [75,76,77,84,85]. The anti-plasticization effect of ROS was similar to that reported for atorvastatin in co-amorphous systems with irbesartan, where atorvastatin increased *T*_g_ and enhanced the physical stability of amorphous irbesartan [16].

Stability studies demonstrated that ROS AM is highly resistant to physical ageing, showing no changes after storage at 45 °C for 1440 min, and remained amorphous after storing at 45 °C for three years, the same as its co-amorphous system with CAR in a 1:1 molar ratio. This stability is notable given the fragile nature of ROS, as indicated by its high fragility parameter (*m* = 97) and activation energy of structural relaxation *E*_a_ = 734.7 kJ·mol^−1^.

Overall, the results indicate that rosuvastatin readily forms stable amorphous and co-amorphous systems (with carvedilol). The favourable miscibility, absence of chemical incompatibility, and long-term stability support the potential of CAR–ROS co-amorphous systems as promising candidates for fixed-dose combination formulations. Further studies evaluating dissolution behaviour and biopharmaceutical performance are required to confirm their therapeutic potential.

## 5. Conclusions

The physicochemical characterisation of rosuvastatin and its co-amorphous system with carvedilol was conducted. Subtle differences between ROS AR and ROS AM forms were detected by thermal methods and SAXS, indicating differences in their structural organisation. These findings highlight the influence of preparation history on the physicochemical properties of the amorphous phase and are consistent with kinetically generated polyamorphic behaviour (pseudopolyamorphism). The ability of TMDSC to separate reversing and non-reversing contributions proved critical for distinguishing between ROS AR and ROS AM. While the reversing glass-transition signal remained essentially unchanged, clear differences were observed in the non-reversing component, indicating that the two forms differ primarily in their degree of structural relaxation and thermal history rather than in their glass-transition behaviour.

In addition to the thermal and structural characterisation, dynamic vapour sorption analysis was performed to assess the surface properties and hygroscopic behaviour of the amorphous rosuvastatin as received. The AR sample of rosuvastatin exhibited a small surface area available for adsorption, but with a tendency to adsorb water in multilayers and absorb it in the bulk.

ROS AM exhibits a relatively high fragility parameter (*m* = 97); however, it remained amorphous even after prolonged storage. This apparent contradiction may be explained by its high glass transition temperature (~122 °C), which markedly reduces molecular mobility at storage temperatures. Thus, the stabilising effect of the high *T*_g_ appears to outweigh the potential destabilising influence associated with the fragile glass-forming nature of the material.

ROS co-amorphous systems with carvedilol were successfully obtained. Thermal analysis revealed non-ideal mixing behaviour in co-amorphous systems; however, the nature of the molecular interactions responsible for this behaviour remains unclear and requires further investigation.

Ageing studies of rosuvastatin showed no difference after storage at 45 °C for 1440 min; it also remained amorphous after three years of storage at the same temperature. The co-amorphous system of carvedilol and rosuvastatin in a 1:1 molar ratio stored at the same temperature over 3 years showed increasing energy of relaxation, but no sign of crystallisation. The obtained co-amorphous systems represent a promising approach for improving the stability of BCS class II drugs. Given that co-amorphous systems have been reported to enhance the dissolution behaviour and bioavailability of poorly soluble drugs, further studies are necessary to evaluate the dissolution kinetics and therapeutic applicability of the obtained co-amorphous system.

## Figures and Tables

**Table 1:** The glass transition temperature (*T*_g_) dependence on heating rate (*q*).

*q* [K·min^−1^]	*T*_g_ [°C]
1	116.48 ± 0.11
2.5	118.34 ± 0.01
5	119.79 ± 0.08
10	120.76 ± 0.29
15	121.52 ± 0.42
20	122.74 ± 1.15

**Table 2:** Experimental and theoretical melting parameters for carvedilol (CAR CR) and its physical mixtures (PM) with rosuvastatin calcium (ROS AR) determined by standard DSC. n/a—not applicable.

Sample	*T*_fus_ ± SD [°C]	Δ_fus_*h* ± SD [J·g^−1^]	Δ_fus_*h*^theoretical^ [J·g^−1^]	ΔΔ_fus_*h* (Δ_fus_*h −* Δ_fus_*h*^theoretical^) [J·g^−1^]
CAR CR	113.8 ± 0.8	116.4 ± 2.2	n/a	n/a
PM CAR CR-ROS AR 2:1 (mol/mol)	113.5 ± 0.1	50.0 ± 0.7	51.9	−1.9
PM CAR CR-ROS AR 1:1 (mol/mol)	113.1 ± 0.3	27.5 ± 0.3	33.4	−5.9
PM CAR CR-ROS AR 1:2 (mol/mol)	113.1 ± 0.3	18.0 ± 1.7	19.5	−1.5

**Table 3:** Experimental glass transition temperatures (*T*_g_), change in heat capacities (Δ*c_p_*) at the *T*_g_ determined from standard DSC 2nd heating run and *T*_g_ calculated from the Couchman–Karasz equation (*T*_g_^CK^) for investigated samples. n/a—not available, n/d—not determined.

Sample	*T*_g_^Experimental^ ± SD [°C]	Δ*c*_p_ ± SD [J g^−1^]	*T*_g_^CK^ [°C]	Δ*T*_g_ (*T*_g_^Experimental^ − *T*_g_^CK^) [°C]
CAR AM	38.0 ± 0.1	0.82 ± 0.02	n/a	n/a
ROS AR	127.1	n/d	n/a	n/a
ROS AM	121.9 ± 0.3	0.41 ± 0.01	n/a	n/a
COAM CAR-ROS 2:1 (mol/mol)	55.1 ± 1.2	0.57 ± 0.02	70.0	−14.9
COAM CAR-ROS 1:1 (mol/mol)	74.0 ± 1.0	0.49 ± 0.02	84.3	−10.3
COAM CAR-ROS 1:2 (mol/mol)	88.7 ± 0.9	0.39 ± 0.01	97.7	−9.0

## Data Availability

Dataset available on request from the authors.

## Supplementary Materials

The following supporting information can be downloaded at: https://www.mdpi.com/article/10.3390/pharmaceutics18091076/s1, Figure S1: DSC curves of carvedilol (CAR CR) showing (A) 1st heating (solvent evaporation), (B) cooling (glass transition of amorphous material) and (C) 2nd heating (glass transition with enthalpy relaxation of amorphous material). All runs obtained at a 10 °C·min^−1^ heating rate. Endothermic heat flow is shown upwards; Figure S2: ^19^F MAS NMR spectra of as-received rosuvastatin (AR form) and freshly prepared amorphous rosuvastatin (AM form). Spectra recorded at 20 kHz spinning rate at 20 °C. No significant differences are observed for both samples. Asterisks denote spinning sidebands; Figure S3: PXRD of ROS AR after DVS analysis.

## Abbreviations

The following abbreviations are used in this manuscript:

AM	Amorphous
API	Active Pharmaceutical Ingredient
AR	As-received
BCS	Biopharmaceutical Classification System
BET	Brunauer–Emmett–Teller adsorption model
CAR	Carvedilol
CK	Couchman–Karasz equation
CVD	Cardiovascular disease
DSC	Differential Scanning Calorimetry
DTG	Derivative Thermogravimetry
DVS	Dynamic Vapour Sorption
FDC	Fixed-dose combination
FT-IR	Fourier-Transform Infrared (Spectroscopy)
GAB	Guggenheim–Anderson–de Boer adsorption model
NMR	Nuclear Magnetic Resonance
PXRD	Powder X-Ray Diffractometry
ROS	Rosuvastatin
RH	Relative Humidity
SAXS	Small-angle X-ray scattering
SSNMR	Solid-state Nuclear Magnetic Resonance
TGA	Thermogravimetric analysis
TMDSC	Temperature-modulated Differential Scanning Calorimetry
YN	Young–Nelson model
