# Supplementary material for: Relaxation Polyamorphism of Rosuvastatin and Physicochemical Characterisation of a Rosuvastatin–Carvedilol Co-Amorphous System
Source: Pharmaceutics. 2026 Aug 27;18(9):1076. doi: 10.3390/pharmaceutics18091076 (PMC13610916; doi:10.3390/pharmaceutics18091076)
Supplement: Supplementary file 1 [file pharmaceutics-18-01076-s001.zip › pharmaceutics-4452321-supplementary.pdf]

SUPPLEMENTARY MATERIAL

Relaxation polyamorphism of rosuvastatin and physicochemical characterisation of a rosuvastatin–carvedilol co-amorphous system

Agata Olszewska[1,2], Maria Brycka[1], Anita Umerska[3], Lidia Tajber[3], Marek Pyda[4,5†] and Marcin Skotnicki[1,3*]

[1] Industrial Pharmacy Division, Chair and Department of Pharmaceutical Technology, Poznan University of Medical Sciences, Poznan, Poland; agata.olszewska@ump.edu.pl (A.O.); (M.B.);
[2] Doctoral School, Poznan University of Medical Sciences, Poznan, Poland; agata.olszewska@ump.edu.pl (A.O.);
[3] School of Pharmacy and Pharmaceutical Sciences, Trinity College Dublin, The University of Dublin, College Green, Dublin 2, D02 PN40, Dublin, Ireland umerskam@tcd.ie (A.U.); lidia.tajber@tcd.ie (L.T.);
[4] Department of Chemistry, University of Tennessee, Knoxville, TN 37996-1600, USA;
[5] Chemical Sciences Division, Oak Ridge National Laboratory, Oak Ridge, TN 37831-6197, USA; mpyda@utk.edu (M.P.);
[†] retired
[*] Correspondence: marcskot@ump.edu.pl

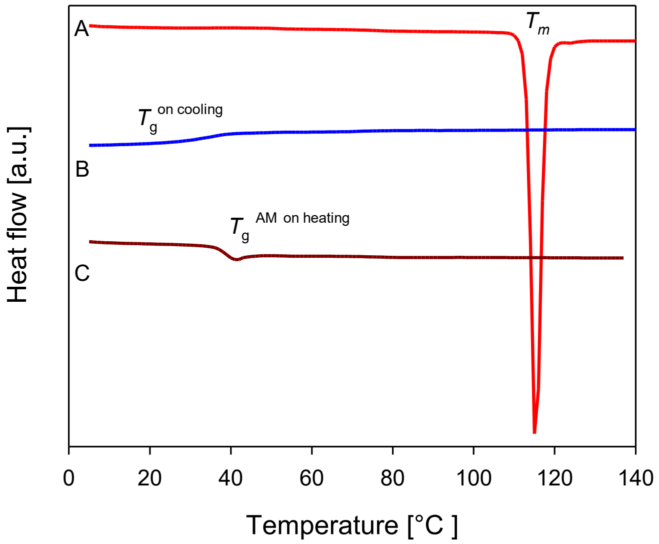

Figure S1: DSC curves carvedilol (CAR CR) showing A) 1st heating (solvent evaporation), B) cooling (glass transition of amorphous material) and C) 2nd heating (glass transition with enthalpy relaxation of amorphous material). All runs obtained at a 10 °C·min⁻¹ heating rate. Endothermic heat flow is shown upwards.

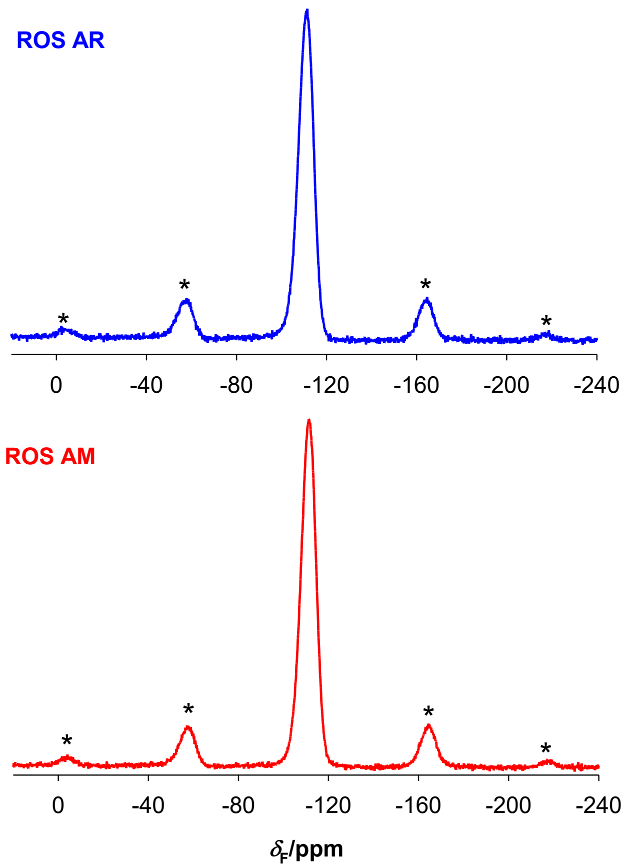

Figure S2: ^{19}F MAS NMR spectra of as-received rosuvastatin (AR form) and freshly prepared amorphous rosuvastatin (AM form). Spectra recorded at 20 kHz spinning rate at 20 °C. No significant differences are observed for both samples. Asterisks denote spinning sidebands.

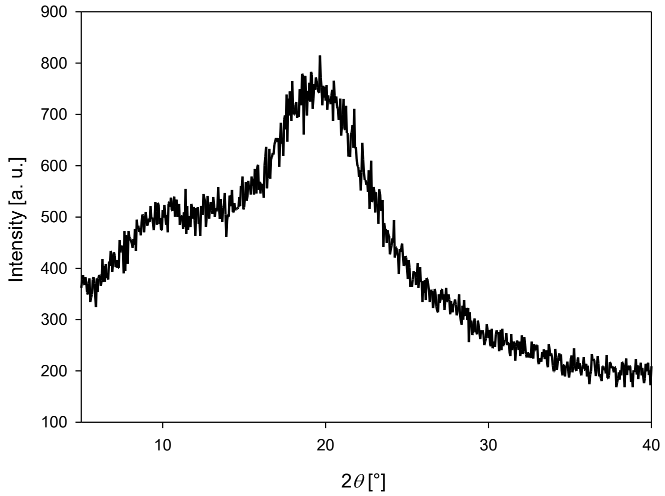

Figure S3: PXRD of ROS AR after a DVS experiment.
